# Palladium-catalyzed borylation of aryl (pseudo)halides and its applications in biaryl synthesis

**DOI:** 10.1186/s13065-018-0510-6

**Published:** 2018-12-19

**Authors:** Hong Ji, Jianghong Cai, Nana Gan, Zhaohua Wang, Liyang Wu, Guorong Li, Tao Yi

**Affiliations:** 1 Key Laboratory of Molecular Target & Clinical Pharmacology, School of Pharmaceutical Sciences & the Fifth Affiliated Hospital, Guangzhou Medical University, Guangzhou, 511436 People’s Republic of China; 2 School of Basic Sciences, Guangzhou Medical University, Guangzhou, 511436 People’s Republic of China; 3 School of Chinese Medicine, Hong Kong Baptist University, Hong Kong, 999077 Hong Kong Special Administrative Region People’s Republic of China

**Keywords:** Palladium-catalyzed borylation, Aryl (pseudo)halides, Suzuki–Miyaura cross coupling, Biaryl synthesis

## Abstract

A facile and efficient palladium-catalyzed borylation of aryl (pseudo)halides at room temperature has been developed. Arylboronic esters were expeditiously assembled in good yields and with a broad substrate scope and good functional group compatibility. This approach has been successfully applied to the one-pot two-step borylation/Suzuki–Miyaura cross-coupling reaction, providing a concise access to biaryl compounds from readily available aryl halides. Furthermore, a parallel synthesis of biaryl analogs is accomplished at room temperature using the strategy, which enhances the practical usefulness of this method.

**Electronic supplementary material:**

The online version of this article (10.1186/s13065-018-0510-6) contains supplementary material, which is available to authorized users.

## Introduction

Arylboronic acids and esters are versatile reagents in organic synthesis. They were widely used in C–C, C–O, C–N and C–S bond forming reactions [1, 2], which are essential for the construction of bioactive molecules and organic building blocks. In particular, functionalized arylboronic esters are highly valuable because they are more stable compared with arylboronic acids [3, 4]. The most common method for the synthesis of arylboronic esters is the reaction of trialkyl borates with aryllithium or Grignard reagents. The method has a problem with functional-group compatibility, and additional protection and deprotection steps are usually required [5]. A series of transition-metal-catalyzed methods for the preparation of arylboronic esters have been developed recently [6–8]. Particularly, palladium-catalyzed synthesis of arylboronic esters from aryl halides or pseudo-halides has opened the door for the development of efficient processes. Some improvements have been reported with respect to catalysts [9–20], ligands [12, 21–24], additives [25, 26] and reaction conditions [18, 19, 27]. However, only very few works have been reported until now on the palladium-catalyzed synthesis of arylboronic esters at room temperature from unactivated aryl chlorides [28].

Biaryl and biheteroaryl motifs are important core structures that are found in natural products, drug molecules and functionalized materials [29–31]. The palladium-catalyzed Suzuki–Miyaura cross-coupling reaction of arylboronic acids or esters with aryl halides has become the most common and powerful method to build such structures [28, 32–34]. Since one-pot two-step protocol combining borylation and Suzuki–Miyaura cross coupling steps was reported in 2004 [35], the need to prepare or purchase a boronic acid or ester could be eliminated. Growing efforts has been paid to develop the attractive method. New catalyst systems such as cyclopalladated ferrocenylimine complex [36, 37] and palladium-indolylphosphine complex [23, 38, 39] were reported successively. In 2007, the first example of borylation/cross-coupling protocol from aryl chlorides was reported [28]. With all of the advances, the one-pot two-step protocol still suffers from high catalyst loads, limited substrate scope and poor functional-group tolerance, and requires high temperature and long reaction time.

Herein, we reported a highly practical and efficient method for palladium-catalyzed borylation of aryl halides or pseudo-halides at room temperature. Furthermore, a facile single pot synthesis of biaryl and biheteroaryl compounds via sequential borylation and Suzuki–Miyaura cross coupling reaction was presented. The approach has been successfully applied in formats amenable to parallel synthesis of biaryls.

## Results and discussion

Initial screening of catalytic systems for the Miyaura borylation of 4-chloroanisole (**1a**) were preformed using 2 mol% of palladium catalyst, 3 equiv. of B_2_pin_2_ and 3 equiv. of anhydrous KOAc or K_3_PO_4_. Various palladium catalysts and catalytic systems listed in Table 1 were tested at elevated temperature (Table 1, entries 1–10). Almost no reaction occurred when catalyst Pd(PPh_3_)_4_ [28, 40, 41] or PdCl_2_(dppf) [41] was used (Table 1, entries 1, 4 and 5). PdCl_2_(PPh_3_)_2_ [25, 42] exhibited low activity for borylation of 4-chloroanisole (Table 1, entry 3). Catalytic systems Pd(PPh_3_)_4_/PCy_3_ [43], Pd_2_dba_3_/PCy_3_ [43, 44], Pd_2_dba_3_/XPhos [28, 45], Pd_2_dba_3_/SPhos [28, 45], Pd(OAc)_2_/PCy_3_ [43, 46], Pd(OAc)_2_/XPhos [45, 47] gave moderate to good yields (Table 1, entries 2 and 6–10). Then we tested room temperature for the reaction of 4-chloroanisole. We discovered that these active catalytic systems for the borylation of 4-chloroanisole at elevated temperature were ineffective at room temperature. However, when Pd(OAc)_2_/SPhos [28] which was developed for the borylation of aryl chlorides at lower temperature were employed, the reaction proceeded very slowly, leading to 42% yield of product after 48 h (Table 1, entry 11).

**Table 1:** Pd-catalyzed borylation of 4-chloroanisole (1a) under various conditions


Entry	Catalyst	Solvent	Base	Temp. (°C)	Time (h)	Yield^a^ (%)
1	Pd(PPh_3_)_4_^b^	DMSO	KOAc	80	8	Trace^c^
2	Pd(PPh_3_)_4_/PCy_3_^b^	Dioxane	KOAc	80	8	72^c^
3	PdCl_2_(PPh_3_)_2_^b^	DMF	K_3_PO_4_	80	8	12^c^
4	PdCl_2_(dppf)^b^	DMF	K_3_PO_4_	80	8	Trace^c^
5	PdCl_2_(dppf)^b^	DMSO	KOAc	80	8	Trace^c^
6	Pd_2_dba_3_/PCy_3_^b^	Dioxane	KOAc	110	8	67^c^
7	Pd_2_dba_3_/XPhos^b^	Dioxane	KOAc	110	8	81^c^
8	Pd_2_dba_3_/SPhos^b^	Dioxane	KOAc	110	8	48^c^
9	Pd(OAc)_2_/PCy_3_^b^	Dioxane	KOAc	110	2	69^c^
10	Pd(OAc)_2_/XPhos^b^	Dioxane	KOAc	110	2	76^c^
11	Pd(OAc)_2_/SPhos	Dioxane	KOAc	RT	48	42
12	**9a**	THF	KOAc	RT	2	Trace^d^
13	**9a**	EtOH	KOAc	RT	2	13^d^
14	**9b**	THF	KOAc	RT	2	23^d^
15	**9b**	EtOH	KOAc	RT	2	66^d^
16	**10a**	THF	KOAc	RT	2	21^d^
17	**10a**	EtOH	KOAc	RT	2	12^d^
18	**10b**	THF	KOAc	RT	2	93^d^
19	**10b**	EtOH	KOAc	RT	2	35
20	**10b**	THF	K_3_PO_4_	RT	1	87^e^, 98^f^

Reaction conditions: 4-chloroanisole (**1a**; 1.0 mmol), B_2_pin_2_ (3.0 mmol), base (3.0 mmol), catalyst (2.0 mol%), ligand (4.0 mol%), solvent (2 mL)

^a^Isolated yield

^b^No reaction occurred at room temperature

^c^Sealed tube

^d^B_2_pin_2_ (3.0 mmol), precatalyst (2.0 mol%)

^e^B_2_pin_2_ (3.0 mmol), precatalyst (2.0 mol%), K_3_PO_4_ (2.0 mmol)

^f^B_2_pin_2_ (1.2 mmol), precatalyst (1.0 mol%)

**Scheme 1:**
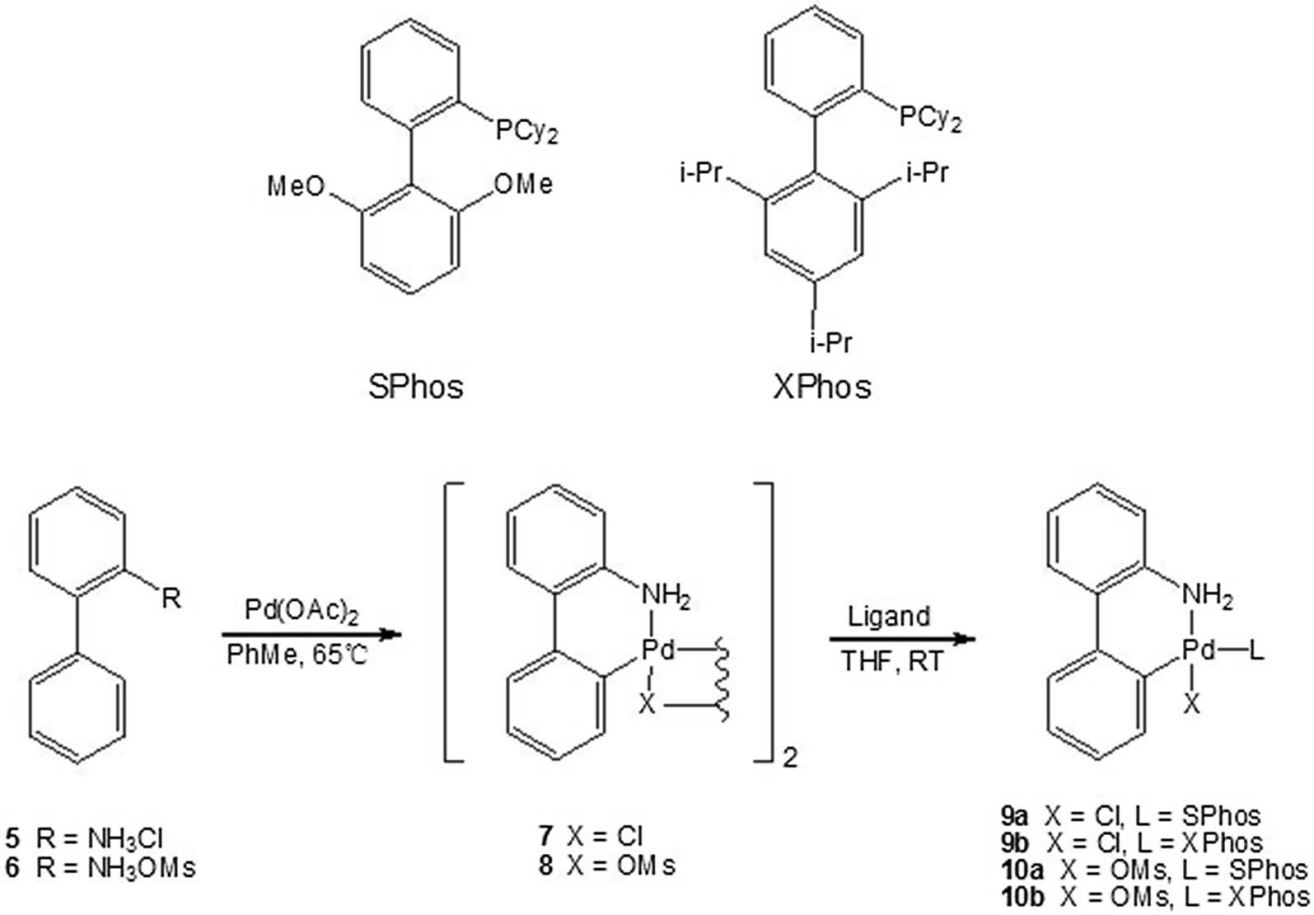
Preparation of precatalyst **9** and **10**

Recently, activated palladium precatalysts have been developed as solutions to the problem of catalyst activation in cross coupling reactions. Many such systems, including pyridine-stabilized NHC precatalysts (PEPPSI) [48], ligated allylpalladium chloride precatalysts [49], imine-derived precatalysts [50] and palladacycle-based precatalysts [34], have been applied to C–C, C-N and C-O bond forming reactions. Since these species are pre-ligated Pd(II) source, some of which can rapidly form a requisite ligated Pd(0) species in situ even at lower temperature when exposed to base [51], we assumed that catalyzed by the species, borylation of aryl halides could proceed in an efficient manner at room temperature. After evaluated a variety of precatalysts, we selected **9** and **10** (Scheme 1), which were more stable in solution and could be readily prepared using commercially available and economical starting materials, as ideal set of precatalysts to test in the borylation reaction. SPhos and XPhos were used as supporting ligands and the μ-Cl and μ-OMs dimmers (**7** or **8**) as palladium sources. Following Buchwald’s protocol [51], the reaction of palladium source μ-Cl or μ-OMs dimmer with ligands rapidly afforded the desired precatalysts **9a**, **9b**, **10a** and **10b** (Scheme 1), which were directly used in our model reaction without isolation, respectively. The results clearly indicated that XPhos is the optimal ligand for this transformation, with the catalyst based on SPhos also showing some activity (Table 1, entries 12–19). Compared with the μ-Cl dimmer (**7**), the μ-OMs (**8**) is optimal as the palladium source. The use of **10b** gave 93% yield of **2a** in THF at room temperature for 2 h (Table 1, entry 18). The results promoted us to optimize the reaction conditions. The effects of solvents, bases and reaction time were examined, and the efficiency of **10b** was further evaluated. In the presence of a sufficient amount of precatalyst (2.0 mol%) and B_2_pin_2_ (3.0 equiv), 2.0 equiv. of K_3_PO_4_ lead to 87% conversion after 1 h, while three equivalents of K_3_PO_4_ gave 98% yield (Table 1, entry 20). Finally, the optimal reaction condition was achieved as the combination of 1.0 mol% **10b**, 1.2 equiv. B_2_pin_2_ and 3.0 equiv. K_3_PO_4_ in THF at room temperature for 1 h (Table 1, entry 20).

In exploring the scope of aryl halides in the borylation reaction, we found that the reaction was broadly amenable to a range of aryl (pseudo)halides with different electronic parameters and bearing a variety of functional groups (Table 2). Electron rich and electron deficient aryl (pseudo)halides were successfully transferred to corresponding boronic esters in good to excellent yields (Table 2, **2b**–**2e** and **2f**–**2m**, 68–98%), as were heteroaromatic halides including indole, thiophene, pyridine and pyrazole (Table 2, **2n**–**2q**, 71–93%). The reaction displayed excellent functional group tolerance and substrates bearing functional groups such as methyl (**2b**), methoxyl (**2c**), phenyl (**2f**), nitrile (**2g**), aldehyde (**2h** and **2j**), trifluoromethyl (**2i**), carboxyl (**2k**), ketone (**2l**) and nitro (**2** **m**) were effective units in the reaction. It is noteworthy that unprotected phenol and aniline also gave the corresponding products **2d** and **2e** in 70% and 84% yields, respectively. No reduced side products were observed in borylation of aldehyde (**2h**, **2j**), ketone (**2l**) and nitro substrate (**2m**). Significantly, besides aryl bromides and iodides, less reactive aryl chlorides and triflates served as effective substrates for this process.

**Table 2:**
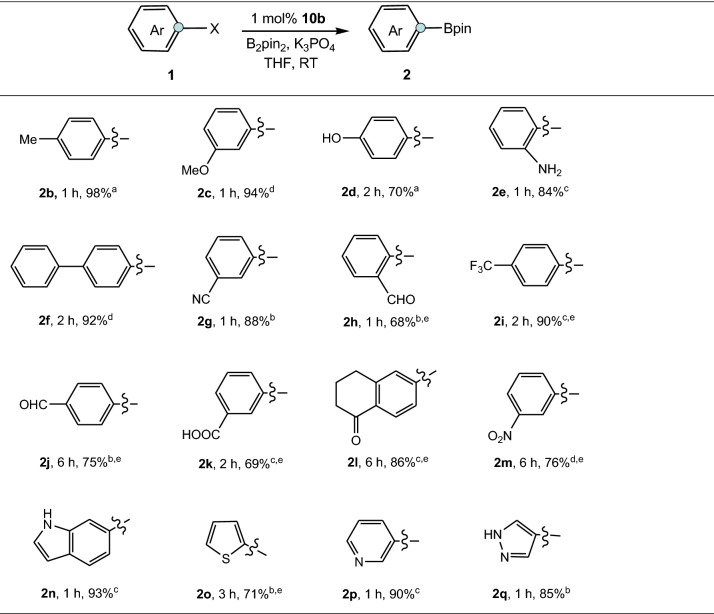
Palladium-catalyzed borylation of aryl (pseudo)halides

Reaction conditions: aryl (pseudo)halide (1.0 mmol), **10b** (1.0 mol%), B_2_pin_2_ (1.2 mmol), K_3_PO_4_ (3.0 mmol), THF (2 mL), RT; isolated yield

^a^X = I

^b^X = Br

^c^X = Cl

^d^X = OTf

^e^**10b** (2.0 mol%)

We subsequently examined a room-temperature tandem borylation/Suzuki–Miyaura coupling procedure to demonstrate the practical utility of the method. The result of borylation of bromobenzene and following coupling with *p*-chlorobenzoic acid proved to be successful under the optimized conditions shown in Table 3. In this process, the aryl halide (**1**) was subjected to Pd-catalyzed borylation conditions with subsequent addition of the aryl halide (**3**) and aqueous K_3_PO_4_. No separation of the boronic ester intermediates was required nor was catalyst added prior to conducting the cross-coupling step. As illustrated by the examples summarized in Table 3, both aryl chlorides and bromides performed well whether used as borylated substrates or electrophilic coupling partners in the reaction. Aryl halides with electron-donating groups such as hydroxyl, alkyl and methoxyl (Table 3, entries 3, 6–8), electron-withdrawing groups such as aldehyde and trifluoromethyl (Table 3, entries 4 and 5) were successfully coupled to various aryl and heteroaryl halides in one-pot to deliver a variety of diaryl compounds in 65–94% yield. The *meta*- and *para*-substituted aryl halides gave excellent to good yields (Table 3, entries 1–5). The *ortho*-substituted aryl halides lead to somewhat lower yields (Table 3, entries 6 and 7). However, 2-bromo-1,3-dimethylbenzene showed less reactivity, affording trace amount of the coupling product. Two methyl groups existing at the *ortho*-position to bromine presumably resulted in an extreme steric hindrance which precluded obtaining expected product. Heteroaryl halides employed as the borylated component or cross-coupling partner often resulted in low yield or no reaction at all in previous protocol [52]. The approach developed herein has been shown to be quite effective for heteroaromatic substrates such as pyridine and pyrazole, providing the desired products in good yield (Table 3, entries 8–10).

**Table 3:**
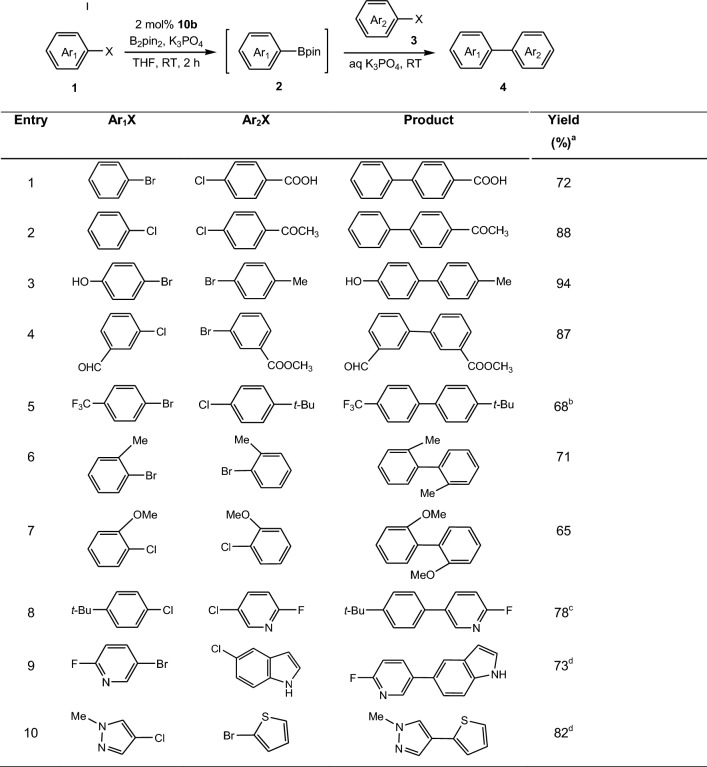
Palladium-catalyzed one-pot two-step preparation of biaryl compounds

Reaction conditions: (a) first halide (1.1 mmol), **10b** (2 mol%), B_2_pin_2_ (1.2 mmol), K_3_PO_4_ (3.0 mmol), THF (4 mL), RT, 2 h; (b) second chloride (1.0 mmol), 3.0 M aq. K_3_PO_4_ (3.0 mmol), RT, 6 h

^a^Yield of isolated product

^b^2 h for the second step

^c^4 h for the second step

^d^10 h for the second step

**Scheme 2:**
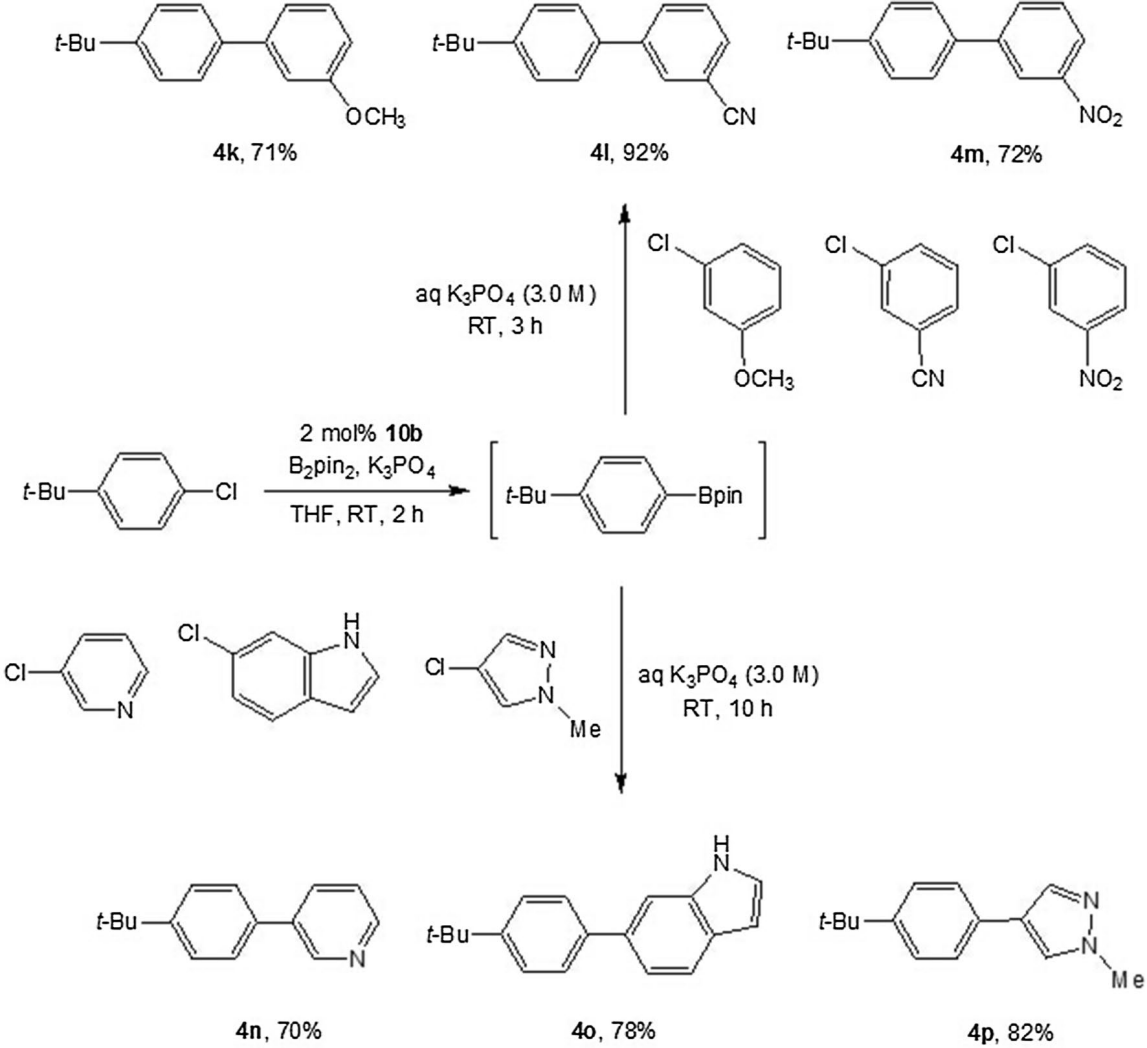
One-pot parallel synthesis of biaryl compounds

Arenes and heteroarenes are frequently present in medicines, agrochemicals, conjugate polymers and other functional materials. To illustrate the practicality of this approach in a medicinal chemistry setting, the chemistry was applied to parallel synthesis of biaryl scaffolds. This allows the preparation of multiple biaryl compounds in parallel from commercial aryl halides in a highly efficient manner. We chose aryl chlorides with polarity differences as electrophile in the second step of the one-opt two-step sequence. An efficient borylation/Suzuki coupling reaction can be performed, affording three distinct products in excellent yields. As shown in Scheme 2, the first chloride 4-*tert*-butyl-1-chlorobenzene was borylated, and the subsequent addition of aqueous K_3_PO_4_ and three aryl chlorides in equimolar amounts provided three desired products (**4k**–**4m**) in 71%, 92% and 72% yield, respectively. Heteroaryl chlorides were also successfully coupled to 4-*tert*-butyl-1-chlorobenzene to yield biaryl compounds (**4n**–**4p**) in good yields.

## Conclusion

In conclusion, we have developed a versatile and efficient protocol for the room-temperature synthesis of arylboronic esters from aryl (pseudo)halides. This method was extended to the one-pot two-step borylation/Suzuki–Miyaura reaction that allowed the coupling of a wide range of aryl halides or heteroaryl halides with excellent functional group tolerance. The precatalyst used in the reaction can be prepared from readily available starting materials in a facile one-pot procedure and can be directly used in the reactions without isolation. The approach also displayed advantages of mild reaction conditions, good stability of catalyst and high efficiency. Further, we successfully applied the approach to parallel synthesis of biaryl compounds, which enable facile preparation of multiple biaryl analogues in a highly efficient manner from readily accessible aryl chlorides at room temperature.

## Additional file

**Additional file 1.** Supporting Informations.
