# Supplementary material for: Palladium-catalyzed borylation of aryl (pseudo)halides and its applications in biaryl synthesis
Source: Chem Cent J. 2018 Dec 19;12:136. doi: 10.1186/s13065-018-0510-6 (PMC6768141; doi:10.1186/s13065-018-0510-6)
Supplement: Supplementary file 1 — Additional file 1. Supporting Informations. [file 13065_2018_510_MOESM1_ESM.doc]

**Supporting Information**

**Palladium-catalyzed borylation of aryl (pseudo)halides and its applications in biaryl synthesis**

Hong Ji*^1^, Jianghong Cai^1^, Nana Gan^1^, Zhaohua Wang^2^, Liyang Wu^1^, Guorong Li^1^, and Tao Yi*^3^

^1^Key Laboratory of Molecular Target & Clinical Pharmacology, School of Pharmaceutical Sciences & the Fifth Affiliated Hospital, Guangzhou Medical University, Guangzhou 511436, P. R. China, ^2^School of Basic Sciences, Guangzhou Medical University, Guangzhou 511436, P. R. China, and ^3^School of Chinese Medicine, Hong Kong Baptist University, Hong Kong Special Administrative Region 999077, P. R. China.

Email: [dljih@126.com](mailto:dljih@126.com), etau2000@163.com

* Corresponding author

**Table of contents**

**General information**....................................................................................................**2**

**General procedure for screening of Pd catalysts**......................................................**2**

**General procedure for screening of precatalysts**......................................................**3**

**General procedure for borylation of aryl (pseudo)halides**......................................**4**

**General procedure for preparation of biaryl compounds**.......................................**8**

**Parallel synthesis of biaryl compounds**....................................................................**11**

**^1^H and ^13^C NMR spectra of final compounds**........................................................ **14**

**Reference**....................................................................................................................**47**

**General Information**

All the reactions were carried out under N_2_ atmosphere. Column chromatography was performed using silica gel (200-300 mesh). TLC analysis of reaction mixtures was performed on the glass-backed silica gel sheets (silica gel HG/T2354-92 GF254) and visualized in UV light (254 nm). Melting points were determined by the open capillary tube method and are uncorrected. ^1^H and ^13^C NMR spectra were recorded on Bruker AV Ⅲ (300 MHz) and JOEL ECA-400 spectrometers. ^1^H and ^13^C chemical shifts are given in ppm relative to TMS. The solvent signals were used as references (CDCl_3_ δ_H_ = 7.26 ppm, δ_C_ = 77.0 ppm; DMSO-d_6_ δ_H_ = 2.50 ppm, δ_C_ = 39.5 ppm).

**General procedure for screening of Pd catalysts**

To an oven-dried test tube equipped with a magnetic stir bar and fitted with a Teflon septum was added palladium catalyst (0.02 mmol), ligand (0.04 mmol), B_2_pin_2_ (3.00 mmol), and base (3.00 mmol). It was then evacuated and refilled with nitrogen. This was repeated three times. After solvent (2 ml) was added *via* syringe through the septum, the mixture was stirred for 10 min, followed by the addition of **1a** (142 mg, 1.00 mmol). The septum was then replaced with a Teflon screwcap and the glass tube was sealed. The reaction mixture was stirred at the indicated temperature for the indicated time in Table 1. The reaction solution was diluted with ethyl acetate (4 ml) and filtered through a thin pad of celite (eluting with ethyl acetate) and the eluent was concentrated under reduced pressure. The crude material so obtained was purified *via* column chromatography on silica gel (petroleum ether/ethyl acetate).

**2-(4-methoxylphenyl)-4,4,5,5-tetramethyl-1,3,2-dioxaborolane (2a)^1^**

White solid, m. p. 27-29 ℃. ^1^H NMR (400 MHz, CDCl_3_) *δ* 7.75 (d, *J* = 8.7 Hz, 2H), 6.89 (d, *J* = 8.7 Hz, 2H), 3.82 (s, 3H), 1.33 (s, 12H). ^13^C NMR (101 MHz, CDCl_3_) *δ* 162.10, 136.47, 113.27, 83.51, 55.05, 24.83.

**General procedure for screening of precatalysts**

**Preparation of precatalyst solution^2^**

**Precatalyst solution 9**: To a 20 ml test tube equipped with a magnetic stir bar and fitted with a Teflon septum was added 2-aminobiphenyl hydrochloride (205 mg, 1.00 mmol, 1.00 eq) and palladium acetate (224 mg, 1.00 mmol, 1.00 eq). The tube was evacuated and backfilled with nitrogen. This sequence was repeated three times, after which THF (5 ml) was added. The mixture was stirred at 60 ºC for 1 h before cooling to room temperature at which point a thick precipitate formed. Heptane (2 ml) was added and the slurry aged for 10 minutes. The solid was collected by filtration and washed with 2:1 THF/heptane (2 x 2 ml) then MeOH (2 ml), and dried under vacuum for 24 hours to provide the pale yellow solid (**7**). To a 3-necked round-bottomed flask fitted with a mechanical stirrer, nitrogen inlet and addition funnel was added the resulting solid (**7**) and ligand (1.00 mmol, 1.00 eq). The flask was evacuated and refilled with N_2_, and this sequence was repeated three times. THF (100 ml) was then added and the solution was allowed to age for ten minutes, with occasional swirling, before used in the following reaction.

**Precatalyst solution 10**: To a 20 ml test tube equipped with a magnetic stir bar and fitted with a Teflon septum was added 2-ammoniumbiphenyl mesylate (263 mg, 1.00 mmol, 1.00 eq) and palladium acetate (224 mg, 1.00 mmol, 1.00 eq). The tube was evacuated and backfilled with nitrogen. This sequence was repeated three times, after which toluene (5 mL) was added. The mixture was stirred at 50° C until it became milky and off-white in appearance. After cooling to room temperature the suspension was filtered, washed with toluene (5 mL) and diethyl ether (3x5 mL), and dried under vacuum for 24 hours to provide an off-white solid (**8**). To a 3-necked round-bottomed flask fitted with a mechanical stirrer, nitrogen inlet and addition funnel was added the resulting solid (**8**) and ligand (1.00 mmol, 1.00 eq). The flask was evacuated and refilled with N_2_, and this sequence was repeated three times. THF (100 ml) was then added and the solution was allowed to age for ten minutes, with occasional swirling, before used in the following reaction.

**The borylation reaction of 1a**: A test tube equipped with a magnetic stir bar and Teflon septum was charged with B_2_pin_2_ (762 mg, 3.00 mmol) and base (3.00 mmol). It was then evacuated and refilled with nitrogen. This was repeated three times. If THF was used as solvent in the reaction, the aged precatalyst solution (**9** or **10**) in THF (2 ml, 2 mol % Pd) prepared above was added by syringe directly. If EtOH was used as solvent in the reaction, the precatalyst solution (**9** or **10**) in EtOH (2 ml, 2 mol % Pd) was added by syringe, which was prepared by removing THF in the aged precatalyst solution prepared above under reduced pressure, and then dissolving the resulting residue in EtOH (2 ml). The mixture under nitrogen was stirred for 10 min, followed by the addition of **1a** (142 mg, 1.00 mmol). The reaction mixture was stirred at room temperature for the indicated time in Table 1. The reaction solution was diluted with ethyl acetate (4 ml) and filtered through a thin pad of celite (eluting with ethyl acetate) and the eluent was concentrated under reduced pressure. The crude material so obtained was purified *via* column chromatography on silica gel (petroleum ether/ethyl acetate).

**General procedure for borylation of aryl (pseudo)halides**

A test tube equipped with a magnetic stir bar and Teflon septum was charged with B_2_pin_2_ (305 mg, 1.20 mmol) and K_3_PO_4_ (636 mg, 3.00 mmol). It was then evacuated and refilled with nitrogen. This was repeated three times. The aged precatalyst solution (**10b**) in THF (1 ml, 1 mol % Pd) prepared above and THF (1 ml) were added by syringe. The mixture under nitrogen was stirred for 10 min, followed by the addition of **1** (1.00 mmol). The reaction mixture was stirred at room temperature until the reaction was complete as monitored by TLC. The reaction solution was diluted with ethyl acetate (4 ml) and filtered through a thin pad of celite (eluting with ethyl acetate) and the eluent was concentrated under reduced pressure. The crude material so obtained was purified via column chromatography on silica gel (petroleum ether/ethyl acetate).

**2-(4-Methylphenyl)-4,4,5,5-tetramethyl-1,3,2-dioxaborolane (2b)^3^**

White solid, m. p. 53-55 ℃. ^1^H NMR (400 MHz, CDCl_3_) *δ* 7.71 (d, *J* = 7.8 Hz, 2H), 7.19 (d, *J* = 7.7 Hz, 2H), 2.36 (s, 3H), 1.34 (s, 12H). ^13^C NMR (101 MHz, CDCl_3_) *δ* 141.39, 134.73, 128.49, 83.57, 24.79, 21.72.

**2-(3-Methoxyphenyl)-4,4,5,5-tetramethyl-1,3,2-dioxaborolane (2c)^3^**

White solid, m. p. 106-107℃. ^1^H NMR (300 MHz, CDCl_3_) *δ* 7.43 – 7.38 (m, 1H), 7.31 (t, *J* = 5.9 Hz, 2H), 7.01 (ddd, *J* = 8.2, 2.8, 1.2 Hz, 1H), 3.83 (s, 3H), 1.34 (s, 12H). ^13^C NMR (101 MHz, CDCl_3_) *δ* 158.98, 128.88, 127.12, 118.66, 117.82, 83.75, 55.14, 24.80.

**2-(4-**[**Hydroxyl**](http://www.baidu.com/link?url=6q-lfP_Llb0YKPuXuA1k8lL_u_yaGgr5W_36Evz-4-Zyw_DN5UqWOScCA13tDf5EpNciR_eOjbrz5P_i5x3kl8vSst36v9oojuGimez99oi)**phenyl)-4,4,5,5-tetramethyl-1,3,2-dioxaborolane (2d)^3^**

White solid, m. p. 109-110℃. ^1^H NMR (300 MHz, CDCl_3_) *δ* 7.70 (d, *J* = 8.5 Hz, 2H), 6.87 – 6.77 (m, 2H), 1.33 (s, 12H).. ^13^C NMR (101 MHz, CDCl_3_) *δ* 158.78, 136.71, 114.96, 83.79, 24.72.

**2-(2-**[**Amino**](http://www.baidu.com/link?url=6q-lfP_Llb0YKPuXuA1k8lL_u_yaGgr5W_36Evz-4-Zyw_DN5UqWOScCA13tDf5EpNciR_eOjbrz5P_i5x3kl8vSst36v9oojuGimez99oi)**phenyl)-4,4,5,5-tetramethyl-1,3,2-dioxaborolane (2e)^4^**

White solid, m. p. 89-90℃. ^1^H NMR (400 MHz, CDCl_3_) *δ* 7.61 (dd, *J* = 7.4, 1.7 Hz, 1H), 7.21 (ddd, *J* = 8.1, 7.2, 1.7 Hz, 1H), 6.67 (td, *J* = 7.3, 1.0 Hz, 1H), 6.61 – 6.57 (m, 1H), 4.73 (s, 2H), 1.34 (s, 12H). ^13^C NMR (101 MHz, CDCl_3_) *δ* 153.58, 136.73, 132.67, 116.80, 114.70, 83.43, 24.85.

**2-[1,1'-biphenyl]-4-yl-4,4,5,5-tetramethyl-1,3,2-Dioxaborolane (2f)^5^**

White solid, m. p. 79-81℃. ^1^H NMR (300 MHz, CDCl_3_) *δ*8.05 (s, 1H), 7.80 (d, *J* = 7.3 Hz, 1H), 7.69 (d, *J* = 7.8 Hz, 1H), 7.63 (d, *J* = 7.3 Hz, 2H), 7.44 (dd, *J* = 14.8, 7.6 Hz, 3H), 7.34 (d, *J* = 7.1 Hz, 1H), 1.36 (s, 12H). ^13^C NMR (101 MHz, CDCl_3_) *δ* 141.09, 140.50, 133.56, 129.97, 128.61, 128.14, 127.17, 83.81, 24.85.

**3-(4,4,5,5-Tetramethyl-1,3,2-dioxaborolan-2-yl)benzonitrile (2g)^4^**

White solid, m. p. 93-94℃. ^1^H NMR (300 MHz, CDCl_3_) *δ* 8.09 (s, 1H), 8.04 – 7.96 (m, 1H), 7.72 (dt, *J* = 7.8, 1.5 Hz, 1H), 7.48 (d, *J* = 7.6 Hz, 1H), 1.35 (s, 12H). ^13^C NMR (101 MHz, CDCl_3_) *δ* 138.65, 138.28, 134.27, 128.30, 118.71, 111.95, 84.38, 24.76.

**2-(4,4,5,5-Tetramethyl-1,3,2-dioxaborolan-2-yl)-benzaldehyde (2h)^3^**

White solid, m. p. 71-73℃. ^1^H NMR (400 MHz, CDCl_3_) *δ* 10.55 (s, 1H), 7.98 – 7.93 (m, 1H), 7.88 – 7.84 (m, 1H), 7.61 – 7.52 (m, 2H), 1.39 (s, 12H). ^13^C NMR (101 MHz, CDCl_3_) *δ* 194.46, 141.11, 135.34, 132.86, 130.60, 127.75, 84.26, 24.72.

**2-[4-(Trifluoromethyl)phenyl)-4,4,5,5-tetramethyl-1,3,2-dioxaborolane (2i)^6^**

White solid, m. p. 67-68 ℃. ^1^H NMR (300 MHz, CDCl_3_) *δ* 7.91 (d, *J* = 7.6 Hz, 2H), 7.61 (d, *J* = 7.5 Hz, 2H), 1.35 (d, *J* = 1.6 Hz, 12H). ^13^C NMR (101 MHz, CDCl_3_) *δ* 135.01, 124.29, 84.25, 24.82.

**4-(4,4,5,5-Tetramethyl-1,3,2-dioxaborolan-2-yl)-benzaldehyde (2j)^4^**

White solid, m. p. 55-57℃. ^1^H NMR (400 MHz, CDCl_3_) *δ* 10.05 (s, 1H), 7.97 (d, *J* = 7.9 Hz, 2H), 7.87 (d, *J* = 8.0 Hz, 2H), 1.37 (s, 12H). ^13^C NMR (101 MHz, CDCl_3_) *δ* 192.79, 137.95, 135.16, 128.70, 84.30, 24.82.

**3-(4,4,5,5-Tetramethyl-1,3,2-dioxaborolan-2-yl)benzoic acid (2k)^7^**

White solid, m. p. 208-210 ℃. ^1^H NMR (400 MHz, CDCl_3_) *δ* 8.58 (s, 1H), 8.21 (dt, *J* = 7.9, 1.4 Hz, 1H), 8.05 (dd, *J* = 7.3, 1.1 Hz, 1H), 7.49 (t, *J* = 7.6 Hz, 1H), 1.37 (s, 12H). ^13^C NMR (101 MHz, CDCl_3_) *δ* 172.14, 139.98, 136.58, 132.82, 128.73, 127.89, 84.19, 24.86.

**6-(4,4,5,5-Tetramethyl-1,3,2-dioxaborolan-2-yl)-1-Tetralone (2l)^8^**

White solid, m. p. 82-83 ℃. ^1^H NMR (300 MHz, CDCl_3_) *δ* 7.94 (s, 1H), 7.77 (dd, *J* = 18.2, 7.6 Hz, 2H), 3.21 – 3.08 (m, 2H), 2.69 (dd, *J* = 8.2, 3.6 Hz, 2H), 1.37 (s, 13H), 1.26 (s, 2H). ^13^C NMR (101 MHz, CDCl_3_) *δ* 207.61, 154.17, 138.98, 133.18, 122.69, 84.27, 36.33, 29.68, 25.63, 24.84.

**2-(3-Nitrophenyl)-4,4,5,5-tetramethyl-1,3,2-dioxaborolane (2m)^3^**

White solid, m. p. 108-109℃. ^1^H NMR (300 MHz, CDCl_3_): *δ* 8.64 (d, *J* = 1.7 Hz, 1H), 8.30 (ddd, *J* = 8.2, 2.4, 1.1 Hz, 1H), 8.10 (d, *J* = 7.3 Hz, 1H), 7.54 (t, *J* = 7.8 Hz, 1H), 1.37 (s, 12H). ^13^C NMR (101 MHz, CDCl_3_) *δ* 147.65, 140.62, 129.31, 128.70, 125.80, 84.52, 24.78.

[**6-(4,4,5,5-Tetramethyl-1,3,2-dioxaborolan-2-yl)-1H-indole**](https://www.guidechem.com/trade/pdetail2393976.html) **(2n)^9^**

White solid, m. p. 169-171℃. ^1^H NMR (300 MHz, CDCl_3_) *δ* 7.90 (d, *J* = 0.7 Hz, 1H), 7.65 (d, *J* = 7.9 Hz, 1H), 7.55 (dd, *J* = 7.9, 0.8 Hz, 1H), 7.26 (t, *J* = 2.8 Hz, 1H), 6.56 (ddd, *J* = 3.0, 2.0, 0.9 Hz, 1H), 1.37 (s, 12H). ^13^C NMR (101 MHz, CDCl_3_) *δ* 135.50, 130.35, 125.56, 120.02, 118.03, 102.63, 83.54, 24.87.

**4,4,5,5-Tetramethyl-2-(thiophen-2-yl)-1,3,2-dioxaborolan (2o)^3^**

White solid, m. p. 76-78℃. ^1^H NMR (300 MHz, CDCl_3_) *δ* 7.65 (dd, *J* = 5.9, 4.1 Hz, 2H), 7.19 (dd, *J* = 4.6, 3.5 Hz, 1H), 1.35 (s, 12H). ^13^C NMR (101 MHz, CDCl_3_) *δ* 137.12, 132.33, 128.18, 84.03, 24.73.

**3-(4,4,5,5-Tetramethyl-1,3,2-dioxaborolan-2-yl)pyridine (2p)^10^**

White solid, m. p. 102-104℃. ^1^H NMR (400 MHz, CDCl_3_) *δ* 8.95 (s, 1H), 8.67 (dd, *J* = 4.9, 1.9 Hz, 1H), 8.06 (d, *J* = 7.5 Hz, 1H), 7.32 – 7.27 (m, 1H), 1.36 (s, 12H). ^13^C NMR (101 MHz, CDCl_3_) *δ* 155.32, 151.87, 142.19, 123.05, 84.14, 24.78.

**4-(4,4,5,5-Tetramethyl-1,3,2-dioxaborolan-2-yl)-*1H*-pyrazole (2q)^11^**

White solid, m. p. 143-145℃. ^1^H NMR (400 MHz, CDCl_3_) δ 7.91 (s, 2H), 1.34 (s, 12H). ^13^CNMR (101 MHz, CDCl_3_) *δ* 140.27, 83.37, 24.75.

**General procedure for preparation of biaryl compounds**

A test tube equipped with a magnetic stir bar and Teflon septum was charged with B_2_pin_2_ (305 mg, 1.20 mmol), and K_3_PO_4_ (636 mg, 3.00 mmol). It was then evacuated and refilled with nitrogen. This was repeated three times. The aged precatalyst solution (**10b**) in THF (2 ml, 2 mol % Pd) prepared above was added by syringe. The mixture under nitrogen was stirred for 10 min, followed by the addition of the first chloride (1.10 mmol). The reaction mixture was stirred at room temperature for 2 h. At this point the second aryl chloride (1.0 mmol) and aqueous K_3_PO_4_ solution (3.0 M, 1.00 ml, 3.00 mmol) were added to the reaction mixture under nitrogen. The reaction mixture was stirred at room temperature for 6 h. The reaction solution was diluted with ethyl acetate (5 ml) and filtered through a thin pad of celite (eluting with ethyl acetate) and the eluent was concentrated under reduced pressure. The crude material so obtained was purified *via* column chromatography on silica gel (petroleum ether/ethyl acetate).

[**4-Biphenylcarboxylic acid**](javascript:showMsgDetail('ProductSynonyms.aspx?CBNumber=CB2359014&postData3=CN&SYMBOL_Type=A');) **(4a)^12^ (Table 3, entry 1)**

White solid, m. p. 217-219 ℃. ^1^H NMR (400 MHz, DMSO-*d_6_*) δ 8.08 – 8.03 (m, 2H), 7.84 – 7.78 (m, 2H), 7.77 – 7.72 (m, 2H), 7.55 – 7.48 (m, 2H), 7.44 (ddd, *J* = 7.2, 3.8, 1.2 Hz, 1H). ^13^C NMR (101 MHz, DMSO-*d_6_*) δ 167.18 144.32, 139.04, 129.99, 129.65, 129.09, 128.30, 126.90.

**4-Acetylbiphenyl (4b)^13^ (Table 3, entry 2)**

White solid, m. p. 120-121℃. ^1^H NMR (400 MHz, CDCl_3_) δ 8.02 (dd, *J* = 8.3, 1.0 Hz, 2H), 7.67 (dd, *J* = 8.2, 1.0 Hz, 2H), 7.64 – 7.59 (m, 2H), 7.46 (ddd, *J* = 7.0, 6.3, 0.9 Hz, 2H), 7.42 – 7.36 (m, 1H), 2.62 (d, *J* = 1.4 Hz, 3H). ^13^C NMR (101 MHz, CDCl_3_) δ 197.68, 145.69, 139.78, 135.77, 128.87, 128.17, 127.17, 26.61.

**4'-Methyl-[1,1'-biphenyl]-4-ol (4c)^14^ (Table 3, entry 3)**

White solid, m. p. 153-154℃. ^1^H NMR (400 MHz, CDCl_3_) *δ* 7.45 (t, *J* = 8.7 Hz, 4H), 7.23 (d, *J* = 7.9 Hz, 2H), 6.90 (d, *J* = 8.6 Hz, 2H), 4.96 (s, 1H), 2.39 (s, 3H). ^13^C NMR (101 MHz, CDCl_3_) *δ* 154.77, 137.80, 136.39, 133.86, 129.41, 128.15, 126.52, 115.52, 21.06.

**Methyl 3'-formyl-[1,1'-biphenyl]-3-carboxylate (4d)^15^ (Table 3, entry 4)**

White solid, m. p. 101-103℃. ^1^H NMR (400 MHz, CDCl_3_) *δ* 10.03 (s, 1H), 8.07 (dd, *J* = 5.8, 4.0 Hz, 3H), 7.83 (ddd, *J* = 7.9, 4.8, 1.4 Hz, 2H), 7.65 – 7.61 (m, 2H), 7.57 (t, *J* = 7.7 Hz, 1H), 3.88 (s, 3H). ^13^C NMR (101 MHz, CDCl_3_) *δ* 192.05, 166.75, 143.98, 140.93, 136.95, 133.08, 130.27, 129.66, 129.58, 129.49, 128.14, 127.08, 52.22.

**4-(*tert*-Butyl)-4'-(trifluoromethyl)-1,1'-biphenyl (4e)^16^ (Table 3, entry 5)**

White solid, m. p. 133-134℃. ^1^H NMR (300 MHz, CDCl_3_) *δ* 7.68 (s, 4H), 7.58 – 7.46 (m, 4H), 1.37 (s, 9H). ^13^C NMR (101 MHz, CDCl_3_) *δ* 151.35, 144.53, 136.81, 127.19, 126.91, 125.95, 125.66, 125.62, 34.62, 31.30.

**2,2'-Dimethylbiphenyl (4f) ^17^ (Table 3, entry 6)**

White solid, m. p. 90-92℃. ^1^H NMR (300 MHz, CDCl_3_) *δ* 7.28 – 7.18 (m, 6H), 7.14 – 7.07 (m, 2H), 2.08 – 2.02 (m, 6H). ^13^C NMR (101 MHz, CDCl_3_) *δ* 141.56, 135.78, 129.77, 129.25, 127.12, 125.50, 19.80.

**2,2'-Dimethoxybiphenyl (4g)^18^ (Table 3, entry 7)**

White solid, m. p.152-154℃. ^1^H NMR (300 MHz, CDCl_3_) *δ* 7.33 (ddd, *J* = 8.1, 7.5, 1.8 Hz, 2H), 7.26 (d, *J* = 1.8 Hz, 1H), 7.23 (d, *J* = 1.8 Hz, 1H), 7.00 (ddd, *J* = 8.2, 6.5, 2.8 Hz, 4H), 3.77 (s, 6H). ^13^C NMR (101 MHz, CDCl_3_) *δ* 156.97, 131.40, 128.55, 127.75, 120.28, 111.03, 55.62.

**3-[4-(*tert*-Butyl)phenyl]-6-fluoro-pyridine (4h)^19^ (Table 3, entry 8)**

White solid, m. p. 104-106 ℃. ^1^H NMR (300 MHz, CDCl_3_) δ8.41 (d, *J* = 2.5 Hz, 1H), 7.96 (ddd, *J* = 8.4, 7.7, 2.6 Hz, 1H), 7.49 (d, *J* = 1.9 Hz, 4H), 6.99 (ddd, *J* = 8.5, 3.0, 0.5 Hz, 1H), 1.37 (s, 9H). ^13^C NMR (101 MHz, CDCl_3_) δ 163.88, 161.98, 151.32, 145.63, 139.51, 134.65, 133.75, 126.68, 126.08, 109.48, 109.18, 34.59, 31.26.

**6-(6-Fluoropyridin-3-yl)-1*H*-indole (4i)^19^ (Table 3, entry 9)**

White solid, m. p. 69-71℃. ^1^H NMR (300 MHz, CDCl_3_) *δ* 8.47 (dd, *J* = 1.6, 0.7 Hz, 1H), 8.40 (brs, 1H), 8.02 (ddd, *J* = 8.4, 7.7, 2.6 Hz, 1H), 7.73 (d, *J* = 8.2 Hz, 1H), 7.56 (d, *J* = 0.7 Hz, 1H), 7.33 – 7.27 (m, 2H), 7.00 (ddd, *J* = 8.5, 3.0, 0.6 Hz, 1H), 6.60 (ddd, *J* = 3.0, 2.0, 0.9 Hz, 1H). ^13^C NMR (101 MHz, CDCl_3_) *δ* 163.93, 145.75, 139.88, 136.26, 135.97, 130.70, 127.79, 125.39, 121.41, 119.33, 109.51, 109.09, 102.65.

**1-Methyl-4-(thiophen-2-yl)-1*H*-pyrazole (4j)^20^ (Table 3, entry 10)**

White solid, m. p. 64-65℃. ^1^H NMR (300 MHz, CDCl_3_) *δ* 7.66 (s, 1H), 7.54 (s, 1H), 7.16 (dd, *J* = 5.0, 1.2 Hz, 1H), 7.03 (ddd, *J* = 8.5, 4.2, 2.4 Hz, 2H), 3.92 (s, 3H). ^13^C NMR (101 MHz, CDCl_3_) *δ* 136.93, 135.00, 127.56, 126.99, 122.90, 122.37, 117.08, 39.03.

**Parallel synthesis of biaryl compounds**

A test tube equipped with a magnetic stir bar and Teflon septum was charged with B_2_pin_2_ (1.22 g, 4.80 mmol), and K_3_PO_4_ (2.54 g, 12.00 mmol). It was then evacuated and refilled with nitrogen. This was repeated three times. The aged precatalyst solution (**10b**) in THF (9 ml, 2 mol % Pd) prepared above and THF (7 ml) were added by syringe. The mixture under nitrogen was stirred for 10 min, followed by the addition of 4-*tert*-butyl-1-chlorobenzene (744 mg, 4.40 mmol). The reaction mixture was stirred at room temperature for 2 h. At this point aqueous K_3_PO_4_ solution (3.0 M, 4.5 ml, 13.5 mmol) were added to the reaction mixture. The three second aryl chlorides (each 1.4 mmol) were subsequently added one after the other to the same pot under nitrogen. The reaction mixture was stirred at room temperature for the given time in scheme 2. The reaction solution was diluted with ethyl acetate (20 ml) and filtered through a thin pad of celite (eluting with ethyl acetate) and the eluent was concentrated under reduced pressure. The crude material so obtained was purified *via* column chromatography on silica gel (petroleum ether/ethyl acetate).

**3-Methoxy-4'-(*tert*-butyl)-1,1'-biphenyl (4k)^21^**

White solid, m. p. 107-109℃. ^1^H NMR (300 MHz, CDCl_3_) *δ* 7.58 – 7.41 (m, 4H), 7.34 (t, *J* = 7.9 Hz, 1H), 7.21 – 7.14 (m, 1H), 7.14 – 7.09 (m, 1H), 6.88 (ddd, *J* = 8.2, 2.6, 0.9 Hz, 1H), 3.86 (s, 3H), 1.36 (s, 9H). ^13^C NMR (101 MHz, CDCl_3_) *δ* 159.86, 150.40, 142.59, 138.16, 129.65, 126.80, 125.66, 119.54, 112.72, 112.37, 55.23, 34.52, 31.34.

**4'-(*tert*-Butyl)-[1,1'-biphenyl]-3-carbonitrile (4l)^22^**

White solid, m. p. 103-105℃. ^1^H NMR (300 MHz, CDCl_3_) *δ* 7.85 (dd, *J* = 2.3, 1.1 Hz, 1H), 7.83 – 7.77 (m, 1H), 7.60 (dt, *J* = 7.7, 1.4 Hz, 1H), 7.53 (d, *J* = 7.7 Hz, 1H), 7.50 (s, 4H), 1.37 (s, 9H). ^13^C NMR (101 MHz, CDCl_3_) *δ* 151.45, 142.09, 135.75, 131.14, 130.32, 130.28, 129.42, 126.59, 125.97, 118.81, 112.74, 34.50, 31.17.

**4'-(*tert*-Butyl)-3-nitro-1,1'-biphenyl (4m)^23^**

White solid, m. p. 111-112℃. ^1^H NMR (300 MHz, CDCl_3_) *δ* 8.45 (t, *J* = 1.9 Hz, 1H), 8.18 (ddd, *J* = 8.2, 2.3, 1.0 Hz, 1H), 7.91 (ddd, *J* = 7.8, 1.7, 1.0 Hz, 1H), 7.65 – 7.48 (m, 5H), 1.38 (s, 9H).^13^C NMR (101 MHz, ) *δ* 151.69, 148.64, 142.62, 135.63, 132.74, 129.56, 126.73, 126.06, 121.65, 121.63, 34.59, 31.22.

**3-[4-(*tert*-Butyl)phenyl]pyridine (4n)^24^**

White solid, m. p. 87-88℃. ^1^H NMR (300 MHz, CDCl_3_) *δ* 8.85 (d, *J* = 1.7 Hz, 1H), 8.57 (dd, *J* = 4.8, 1.6 Hz, 1H), 7.88 (ddd, *J* = 7.9, 2.3, 1.7 Hz, 1H), 7.52 (d, *J* = 1.9 Hz, 4H), 7.36 (ddd, *J* = 7.9, 4.8, 0.8 Hz, 1H), 1.37 (s, 9H). ^13^C NMR (101 MHz, CDCl_3_) *δ* 151.20, 148.12, 148.07, 136.43, 134.81, 134.14, 126.74, 126.00, 123.47, 34.56, 31.20.

**6-[4-(*tert*-Butyl)phenyl]-1*H*-indole (4o)^25^**

White solid, m. p. 74-76℃. ^1^H NMR (300 MHz, CDCl_3_) *δ* 8.15 (brs, 1H), 7.68 (d, *J* = 8.3 Hz, 1H), 7.65 – 7.54 (m, 3H), 7.51 – 7.43 (m, 2H), 7.39 (dd, *J* = 8.2, 1.6 Hz, 1H), 7.21 (dd, *J* = 3.2, 2.5 Hz, 1H), 6.56 (ddd, *J* = 3.1, 2.0, 0.9 Hz, 1H), 1.37 (s, 9H). ^13^C NMR (101 MHz, CDCl_3_) *δ* 149.52, 139.32, 136.33, 135.34, 126.95, 125.63, 124.66, 120.78, 119.70, 109.32, 102.41, 34.46, 31.39.

**4-[4-(*tert*-Butyl)phenyl]-1-methyl-1*H*-pyrazole (4p)^26^**

White solid, m. p. 68-69℃. ^1^H NMR (300 MHz, CDCl_3_) *δ* 7.74 (s, 1H), 7.57 (s, 1H), 7.40 (d, *J* = 1.1 Hz, 4H), 3.94 (s, 3H), 1.33 (s, 9H). ^13^C NMR (101 MHz, CDCl_3_) *δ* 149.20, 136.59, 129.69, 126.62, 125.64, 125.16, 123.02, 38.92, 34.38, 31.25.

**^1^H and ^13^C NMR spectra of final compounds**

**2-(4-methoxylphenyl)-4,4,5,5-tetramethyl-1,3,2-dioxaborolane (2a)**

**
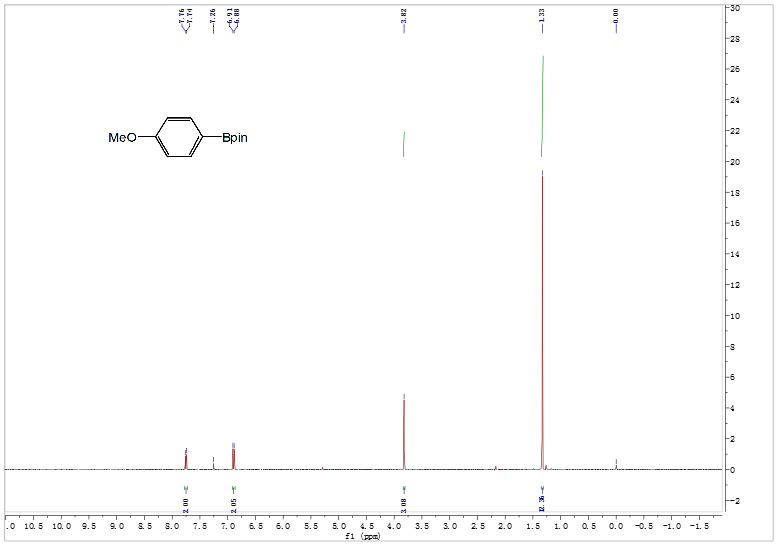
**

**
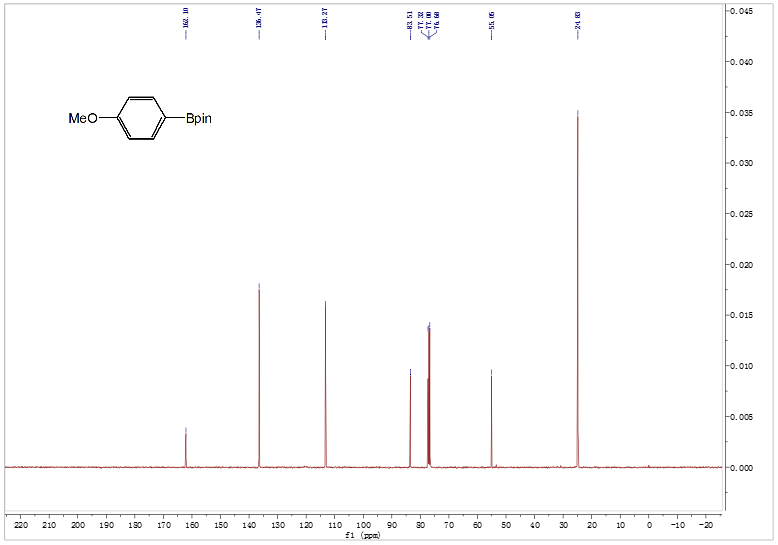
**

**2-(4-Methylphenyl)-4,4,5,5-tetramethyl-1,3,2-dioxaborolane (2b)**

**
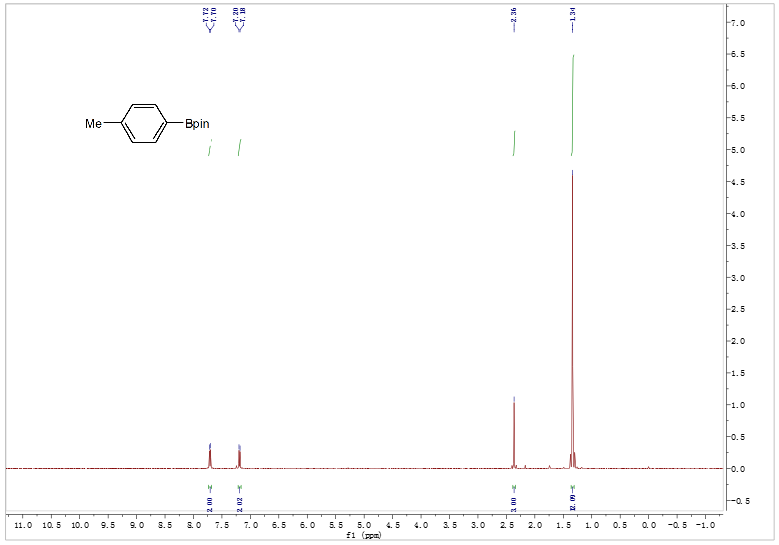
**

**
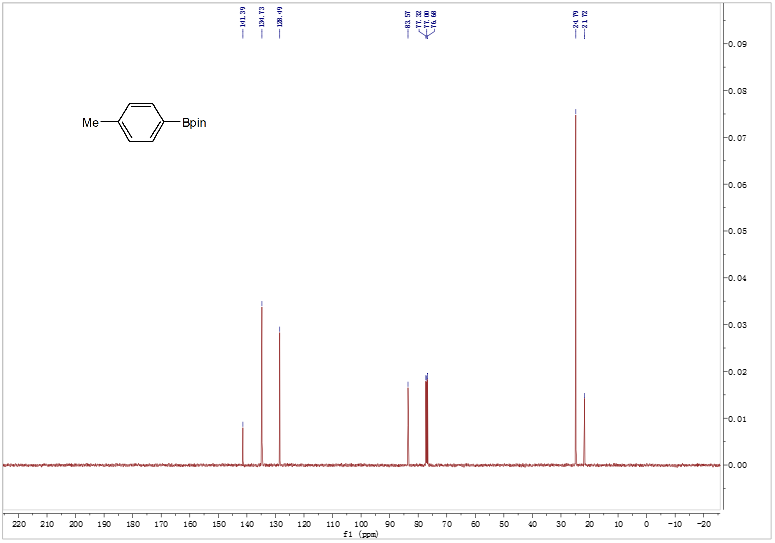
**

**2-(3-Methoxyphenyl)-4,4,5,5-tetramethyl-1,3,2-dioxaborolane (2c)**


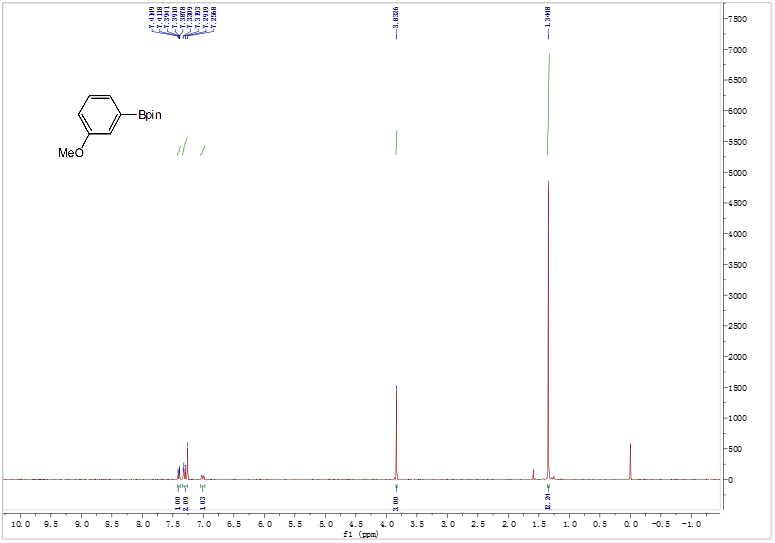


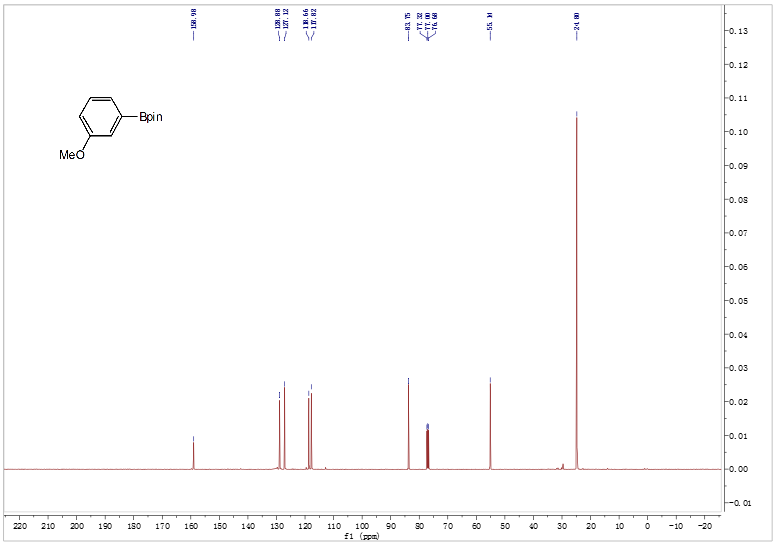


**2-(4-**[**Hydroxyl**](http://www.baidu.com/link?url=6q-lfP_Llb0YKPuXuA1k8lL_u_yaGgr5W_36Evz-4-Zyw_DN5UqWOScCA13tDf5EpNciR_eOjbrz5P_i5x3kl8vSst36v9oojuGimez99oi)**phenyl)-4,4,5,5-tetramethyl-1,3,2-dioxaborolane (2d)**

**
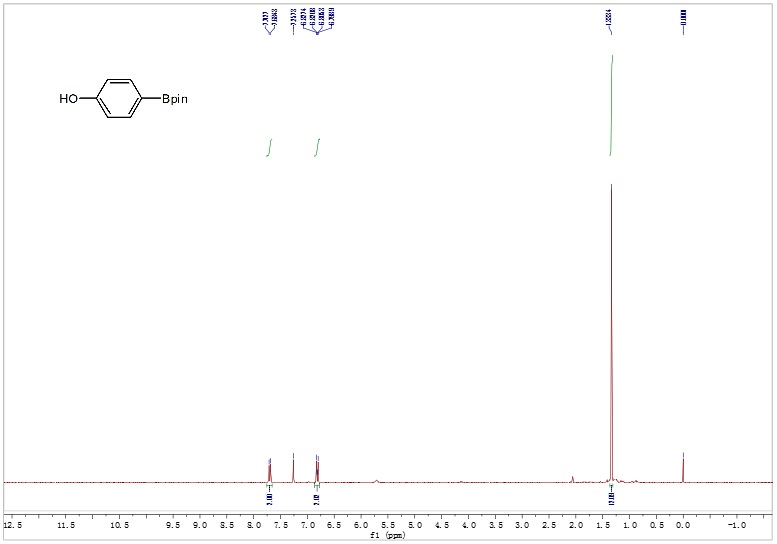
**

**
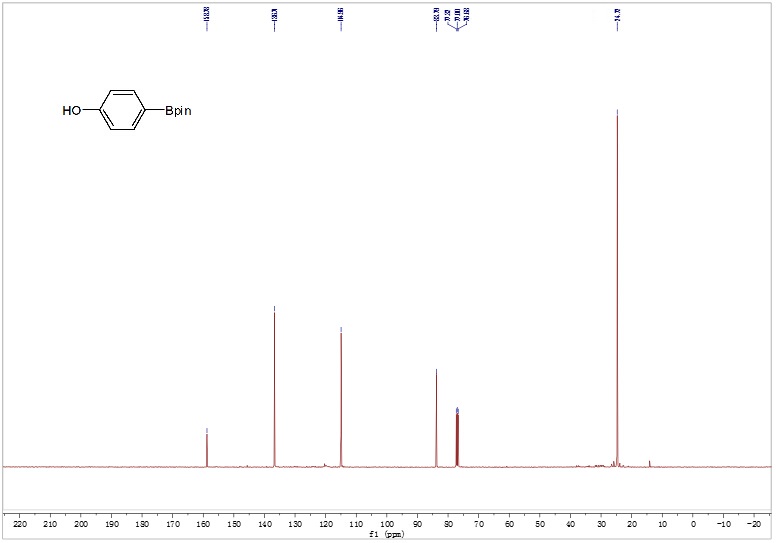
**

**2-(2-**[**Amino**](http://www.baidu.com/link?url=6q-lfP_Llb0YKPuXuA1k8lL_u_yaGgr5W_36Evz-4-Zyw_DN5UqWOScCA13tDf5EpNciR_eOjbrz5P_i5x3kl8vSst36v9oojuGimez99oi)**phenyl)-4,4,5,5-tetramethyl-1,3,2-dioxaborolane (2e)**

**
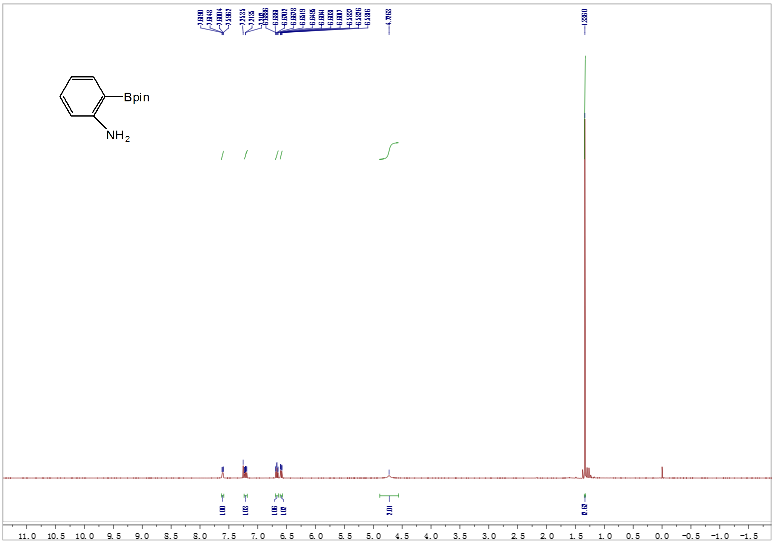
**


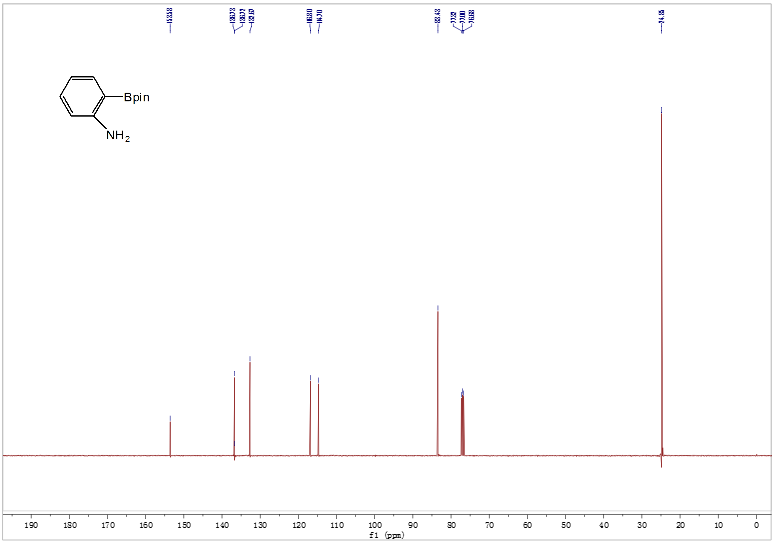


**2-[1,1'-biphenyl]-4-yl-4,4,5,5-tetramethyl-1,3,2-Dioxaborolane (2f)**

**
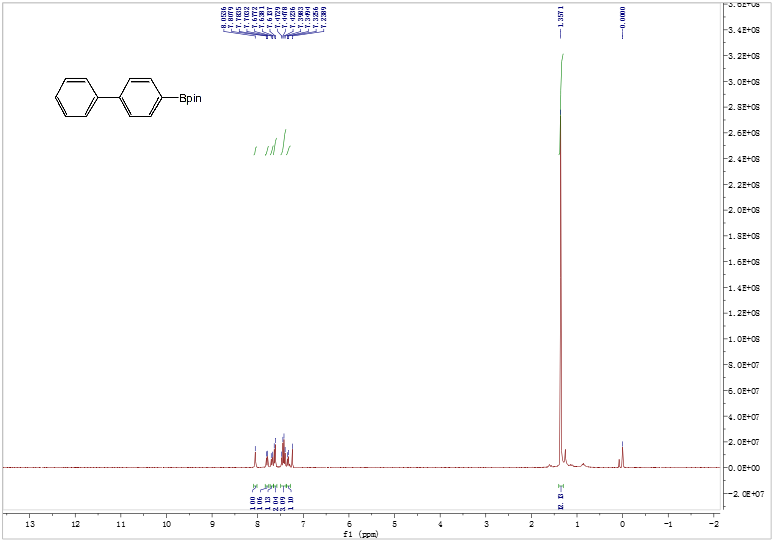
**

**
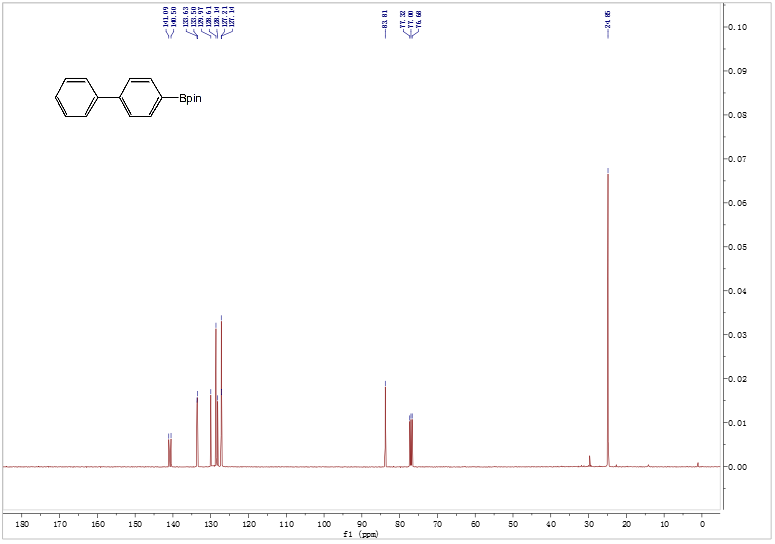
**

**3-(4,4,5,5-Tetramethyl-1,3,2-dioxaborolan-2-yl)benzonitrile (2g)**

**
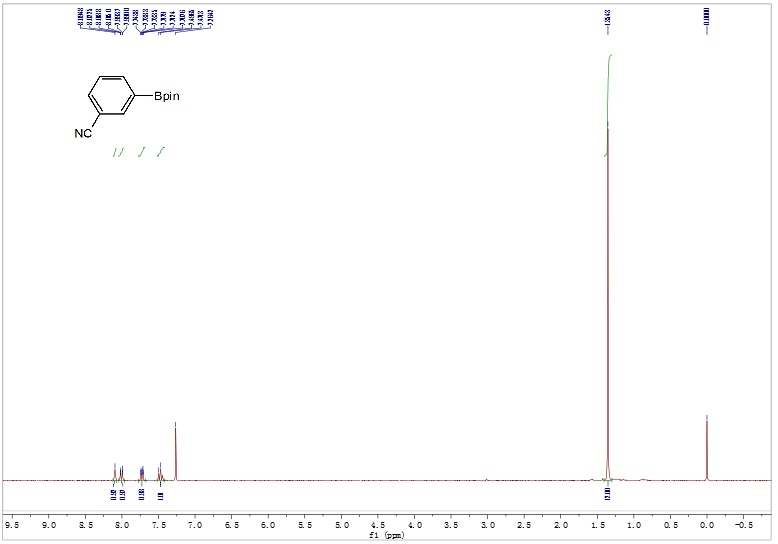
**

**
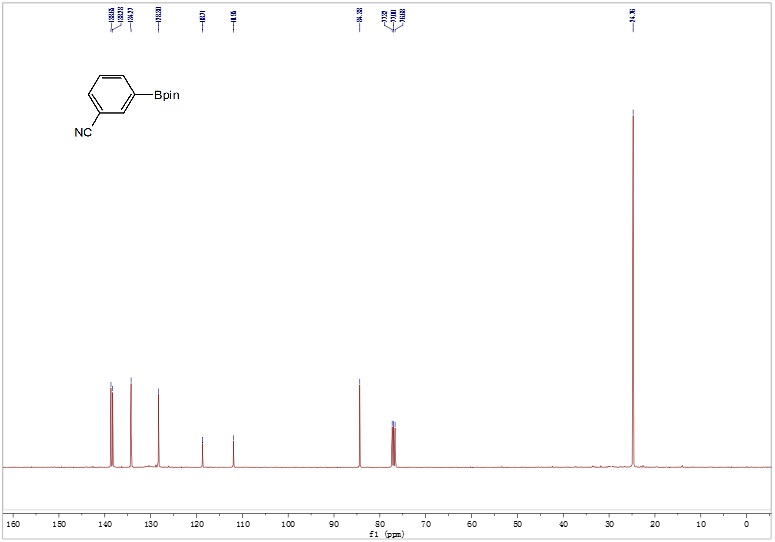
**

**2-(4,4,5,5-Tetramethyl-1,3,2-dioxaborolan-2-yl)-benzaldehyde (2h)**

**
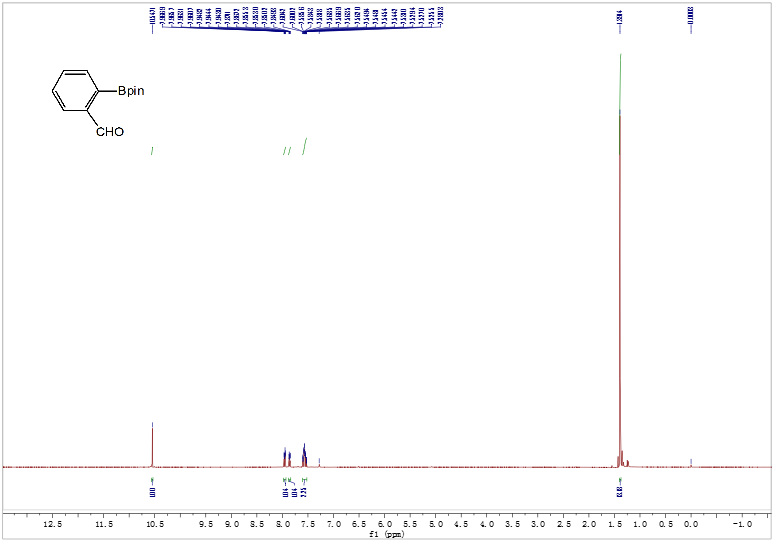
**

**
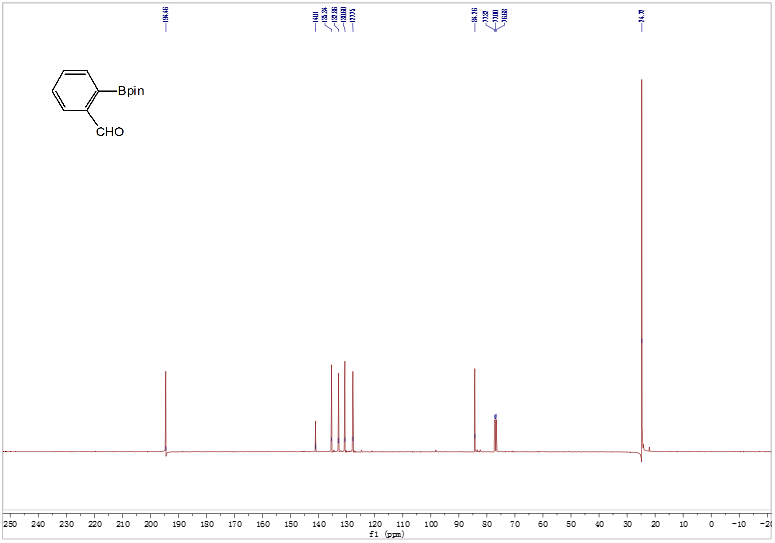
**

**2-[4-(Trifluoromethyl)phenyl)-4,4,5,5-tetramethyl-1,3,2-dioxaborolane (2i)**

**
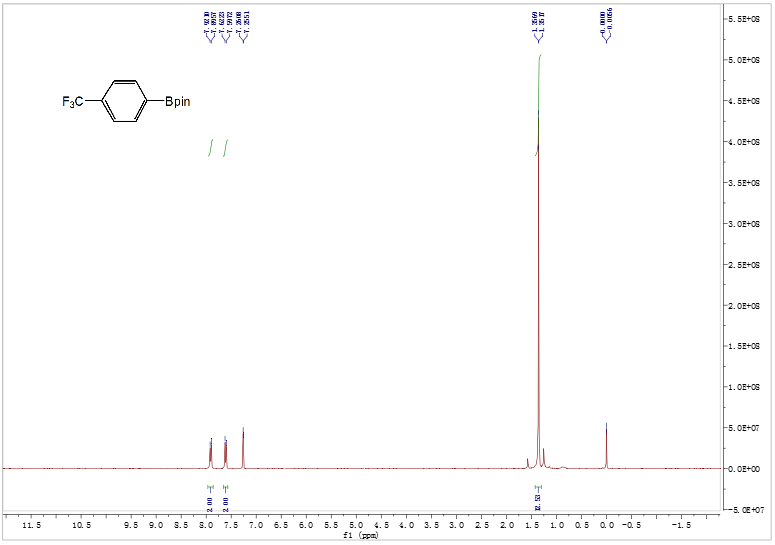
**

**
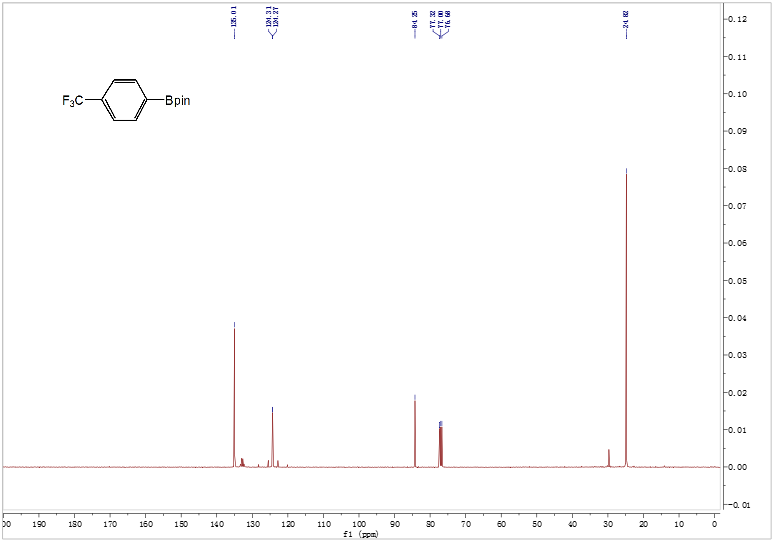
**

**4-(4,4,5,5-Tetramethyl-1,3,2-dioxaborolan-2-yl)-benzaldehyde (2j)**

**
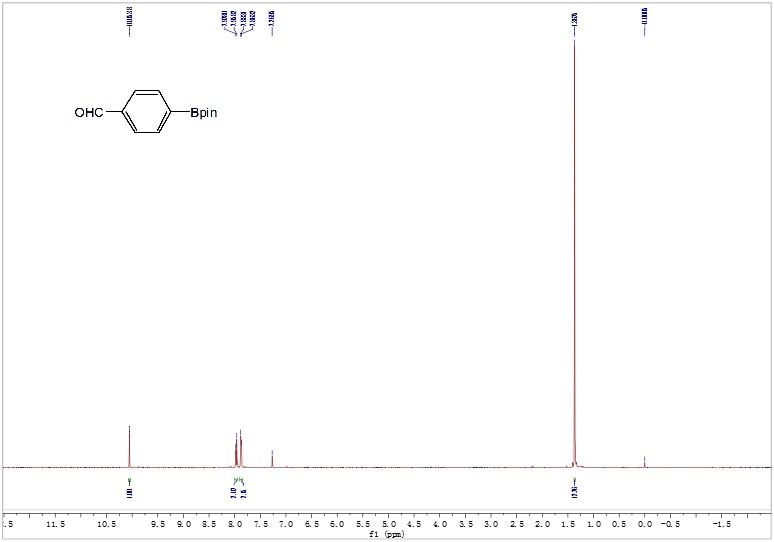
**

**
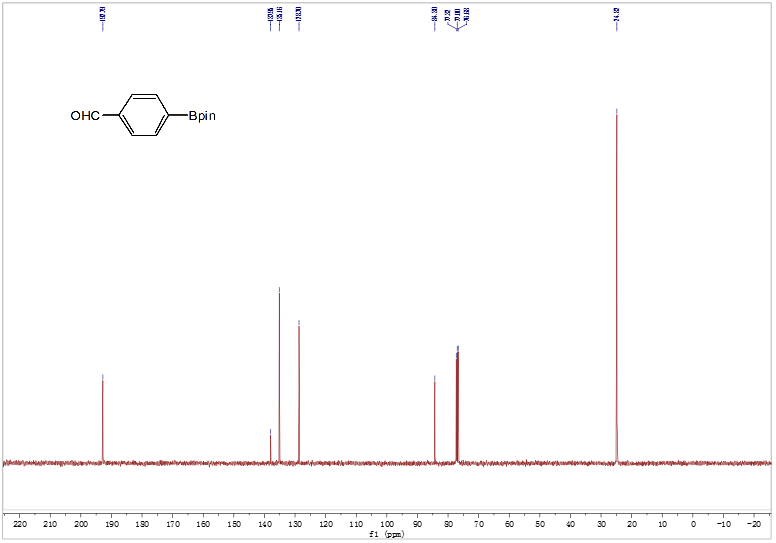
**

**3-(4,4,5,5-Tetramethyl-1,3,2-dioxaborolan-2-yl)benzoic acid (2k)**

**
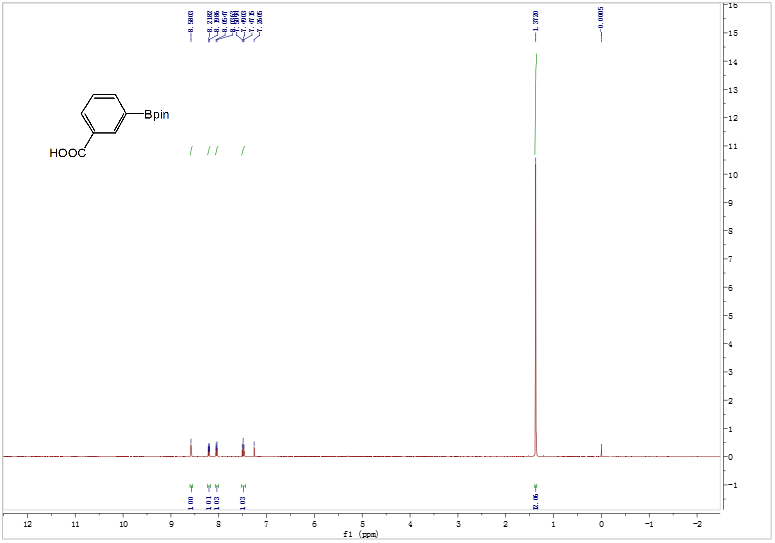
**

**
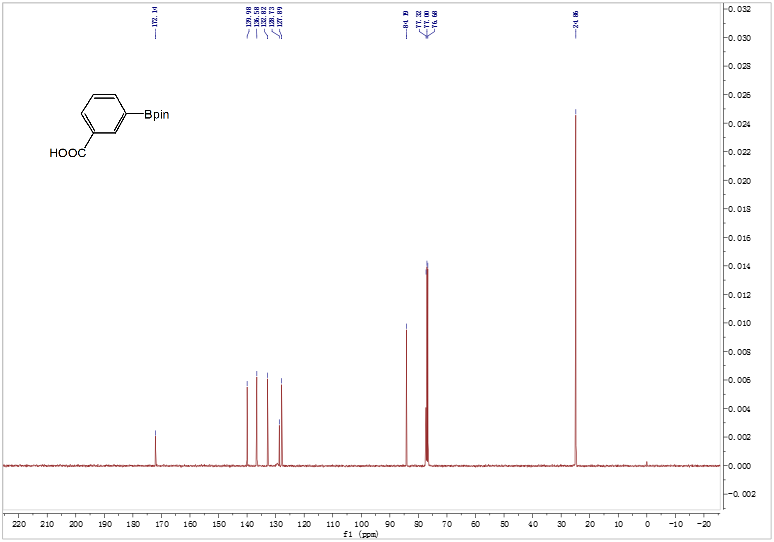
**

**6-(4,4,5,5-Tetramethyl-1,3,2-dioxaborolan-2-yl)-1-Tetralone (2l)**

**
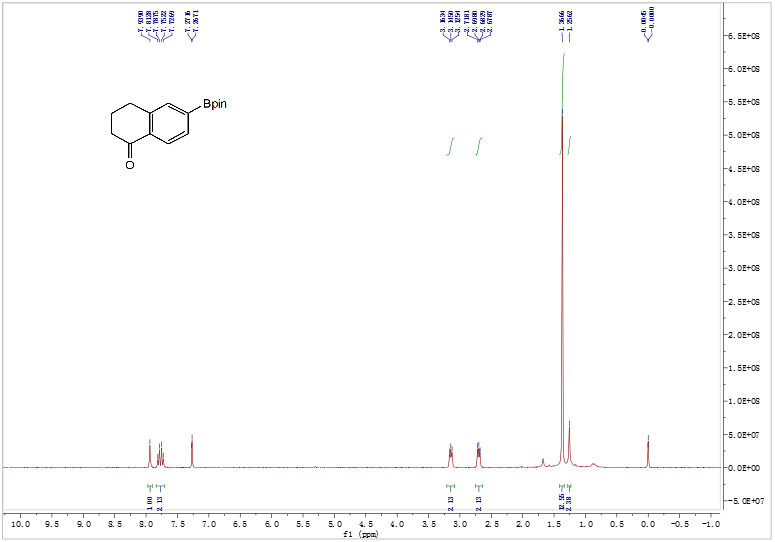
**

**
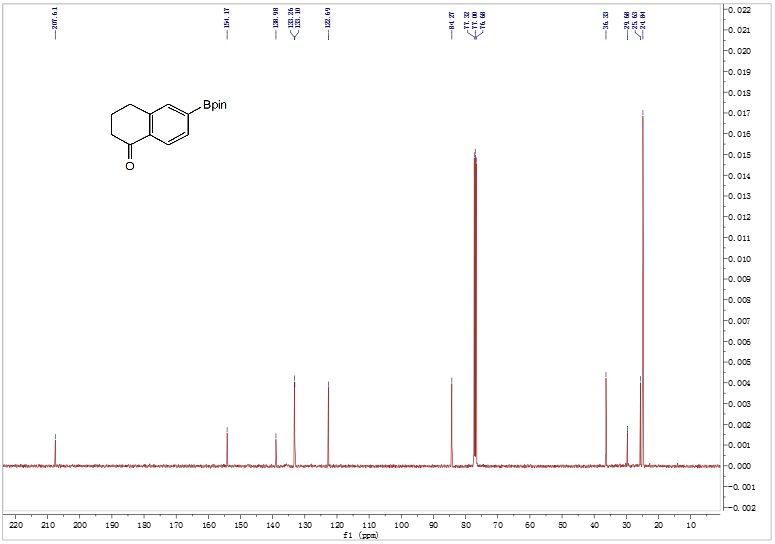
**

**2-(3-Nitrophenyl)-4,4,5,5-tetramethyl-1,3,2-dioxaborolane (2m)**

**
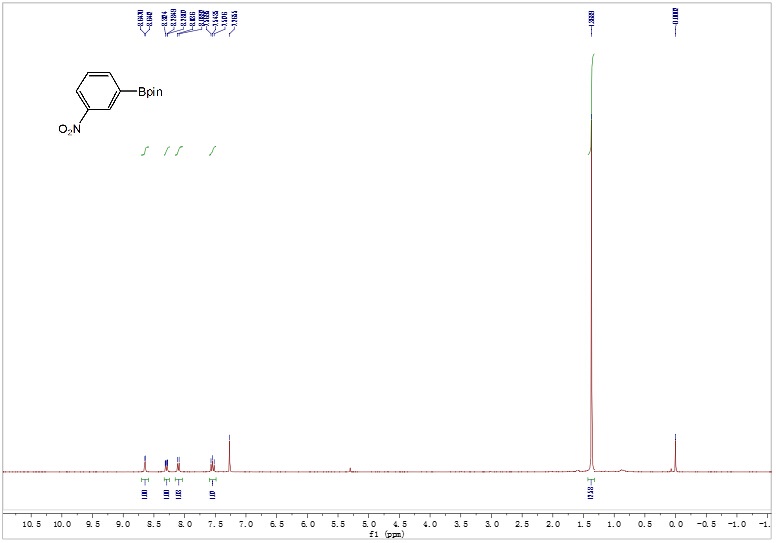
**

**
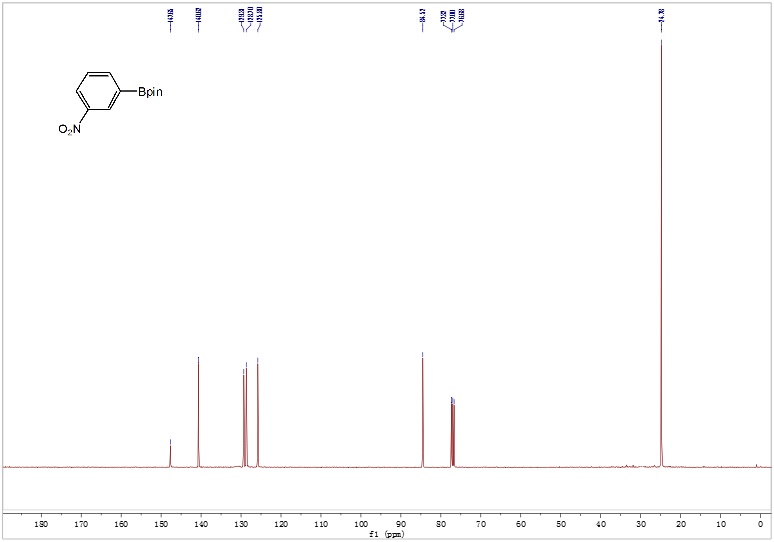
**

[**6-(4,4,5,5-Tetramethyl-1,3,2-dioxaborolan-2-yl)-1H-indole**](https://www.guidechem.com/trade/pdetail2393976.html) **(2n)**

**
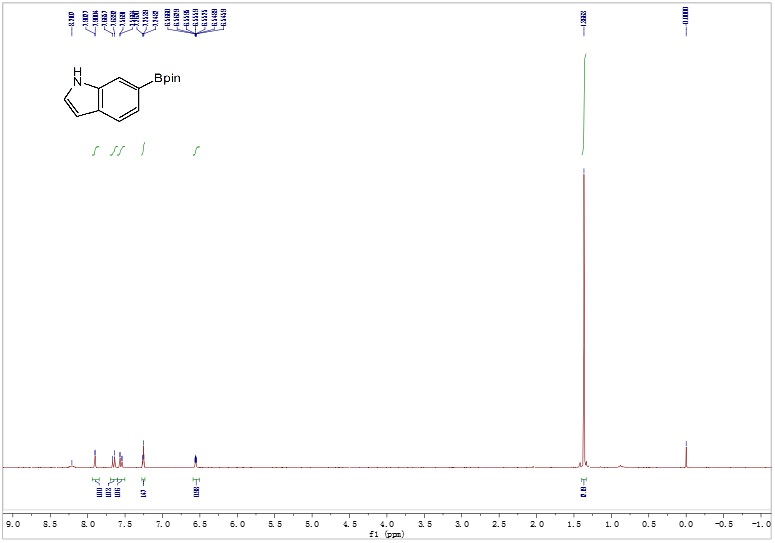
**

**
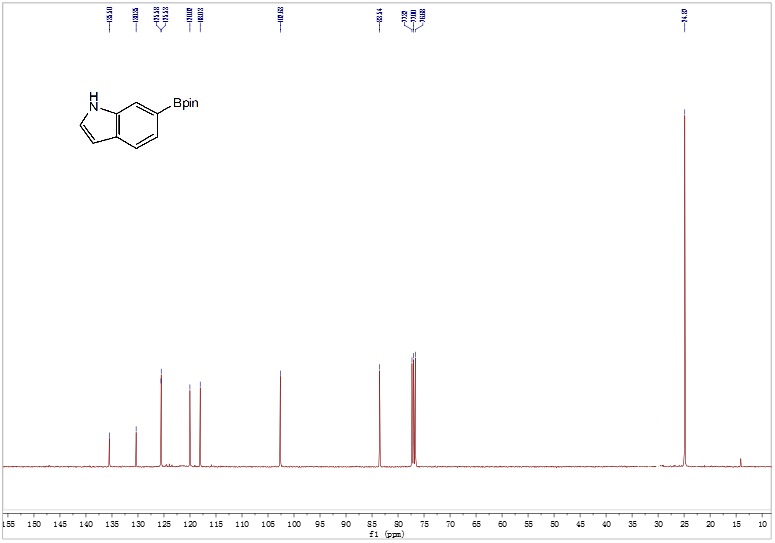
**

**4,4,5,5-Tetramethyl-2-(thiophen-2-yl)-1,3,2-dioxaborolan (2o)**

**
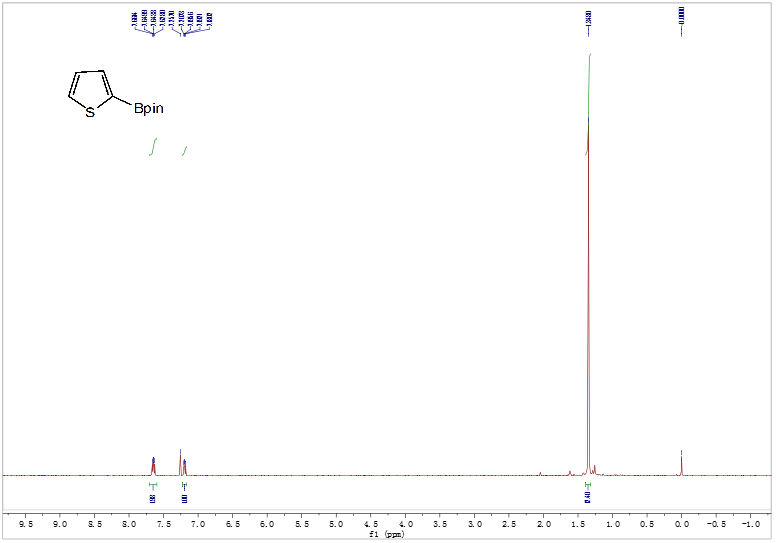
**

**
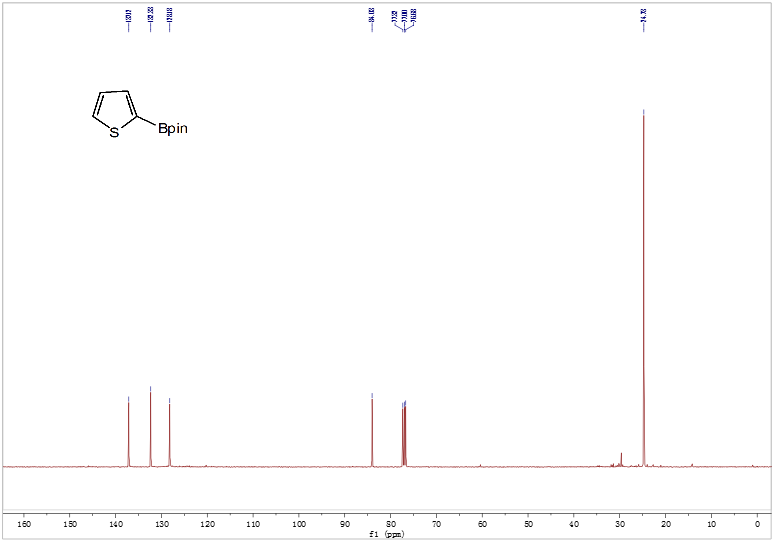
**

**3-(4,4,5,5-Tetramethyl-1,3,2-dioxaborolan-2-yl)pyridine (2p)**

**
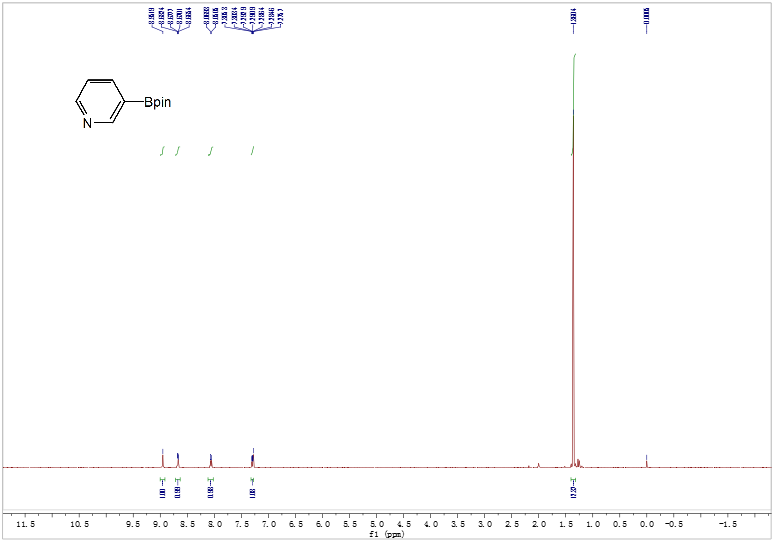
**

**
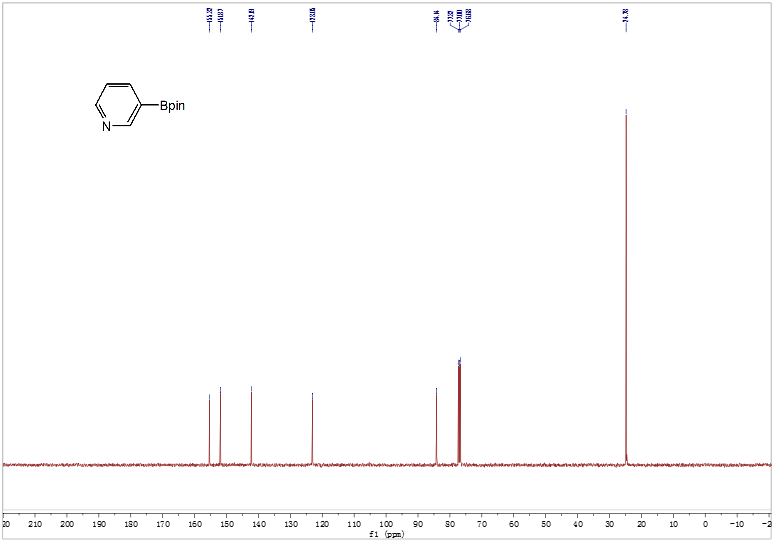
**

**4-(4,4,5,5-Tetramethyl-1,3,2-dioxaborolan-2-yl)-*1H*-pyrazole (2q)**

**
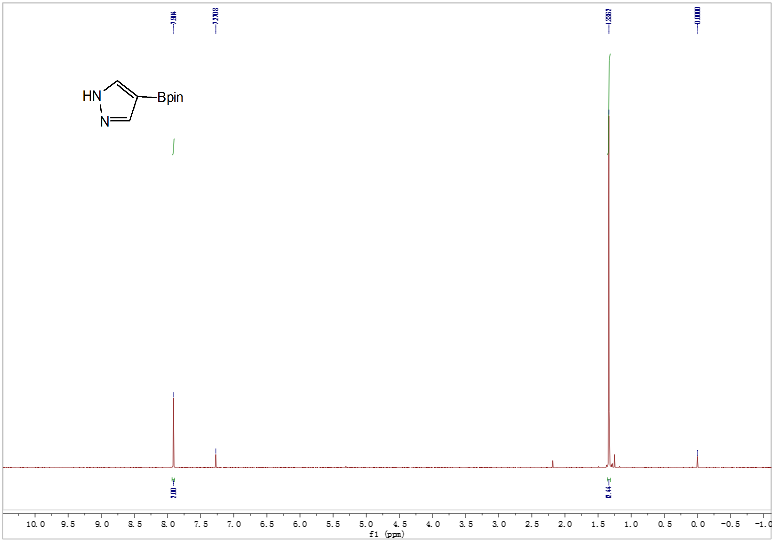
**

**
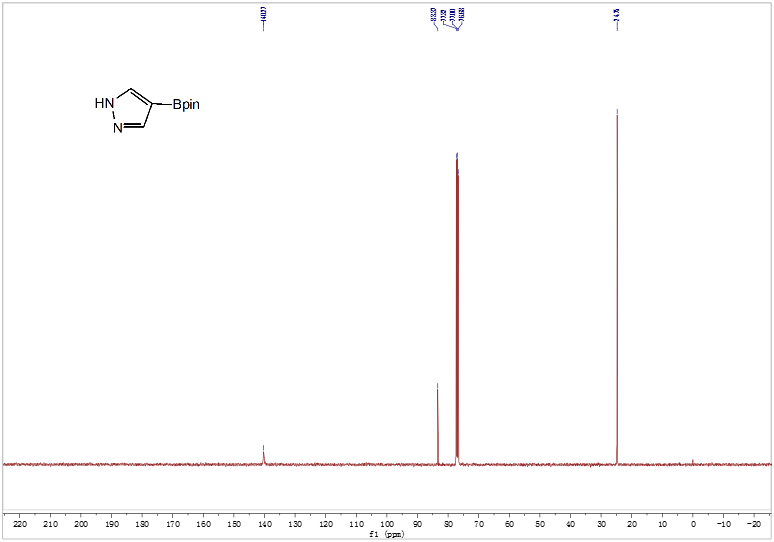
**

[**4-Biphenylcarboxylic acid**](javascript:showMsgDetail('ProductSynonyms.aspx?CBNumber=CB2359014&postData3=CN&SYMBOL_Type=A');) **(4a)**

**
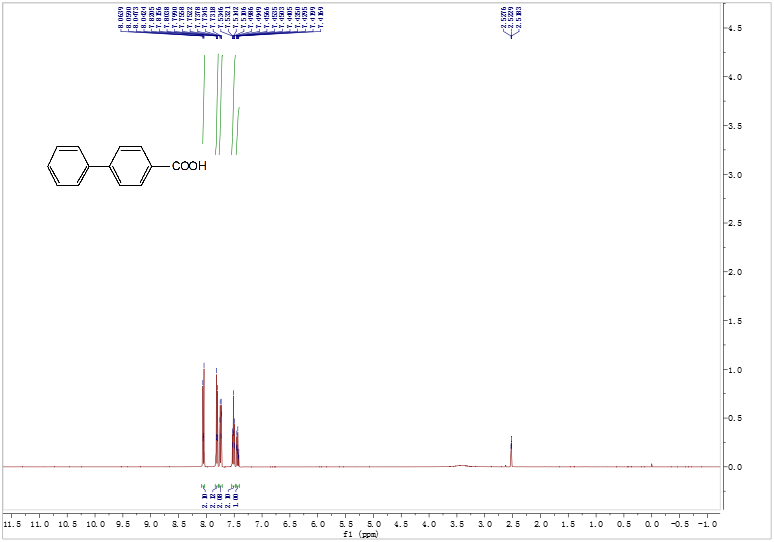
**

**
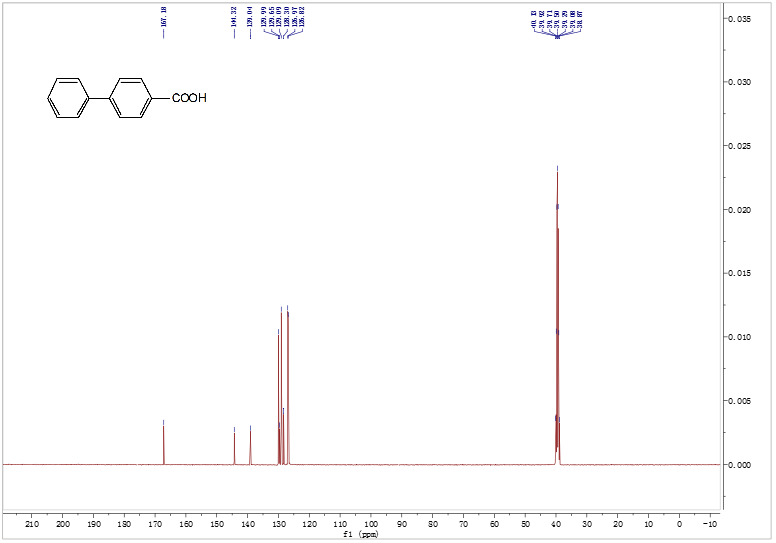
**

**4-Acetylbiphenyl (4b)**

**
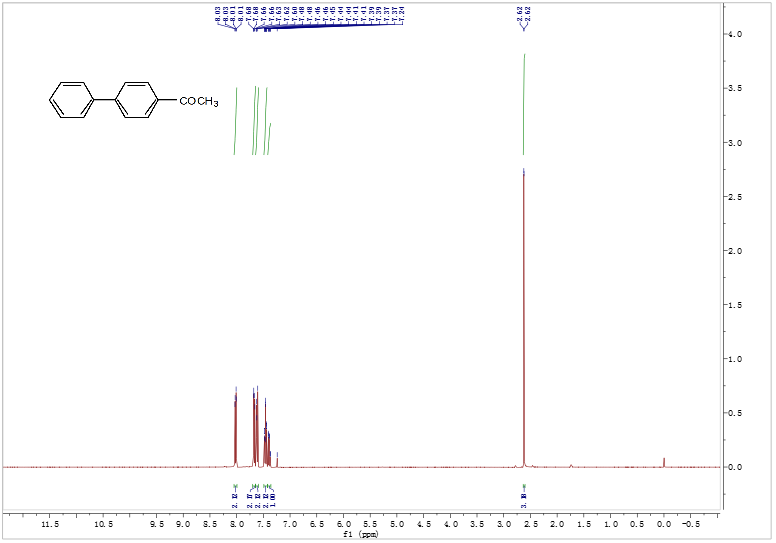
**

**
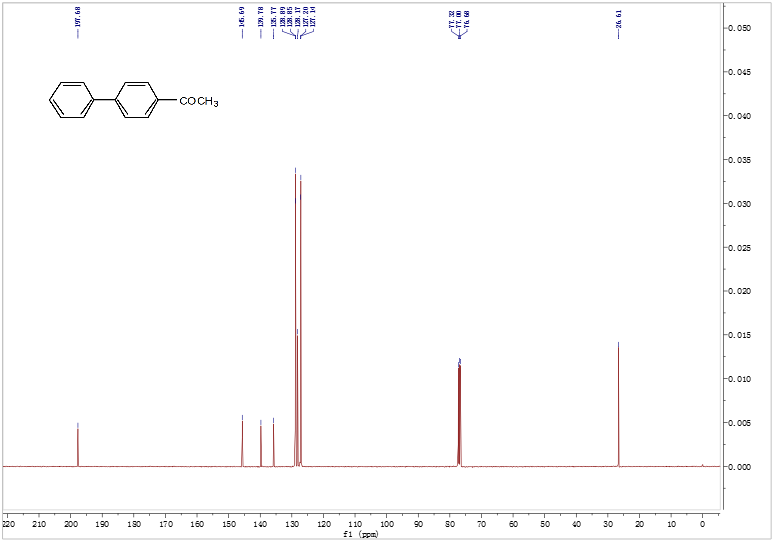
**

**4'-Methyl-[1,1'-biphenyl]-4-ol (4c)**

**
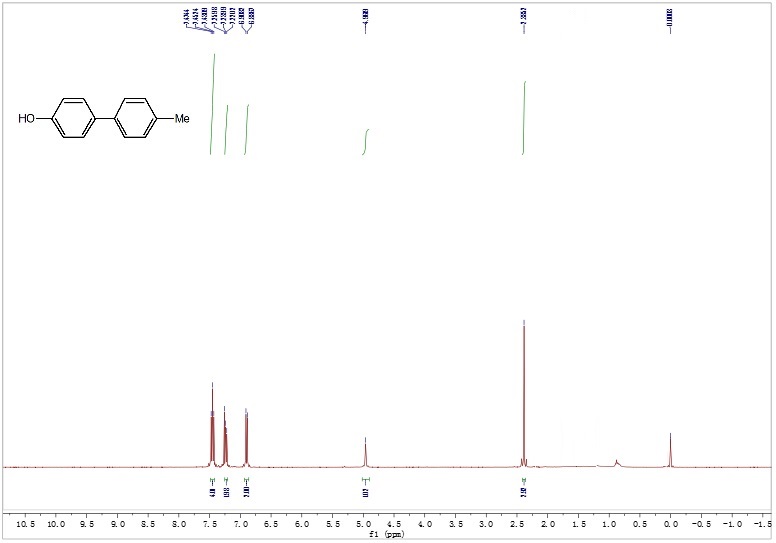
**

**
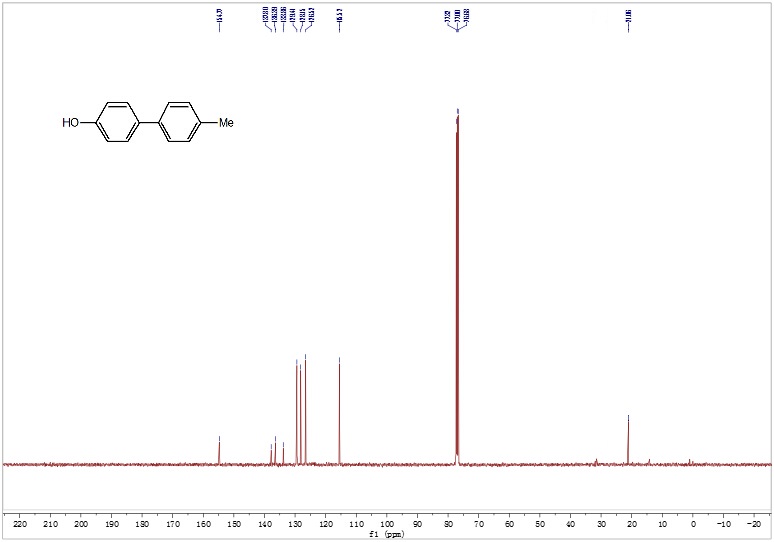
**

**Methyl 3'-formyl-[1,1'-biphenyl]-3-carboxylate (4d)**

**
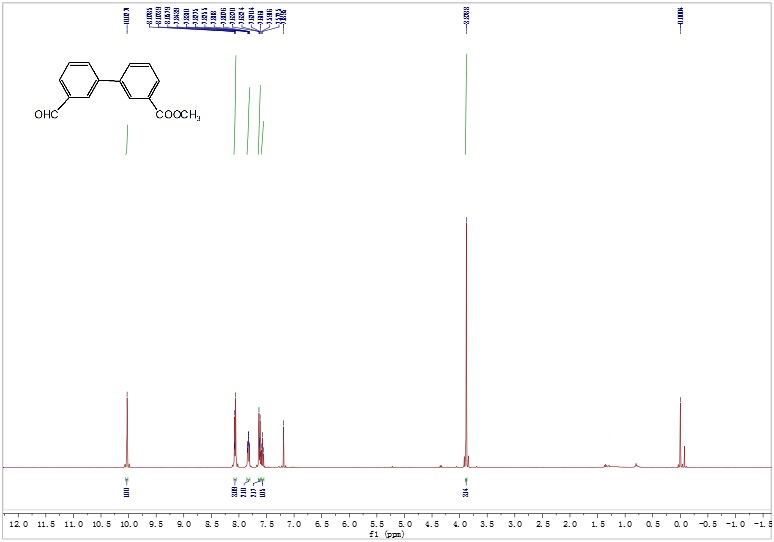
**

**
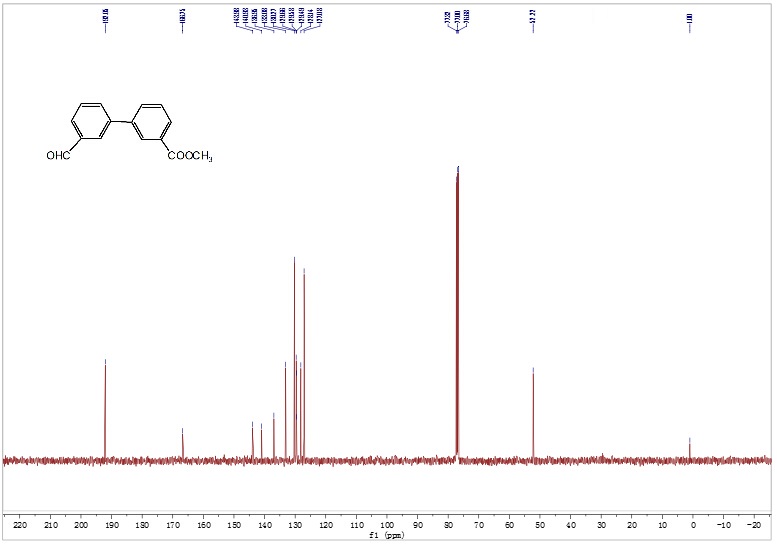
**

**4-(*tert*-Butyl)-4'-(trifluoromethyl)-1,1'-biphenyl (4e)**

**
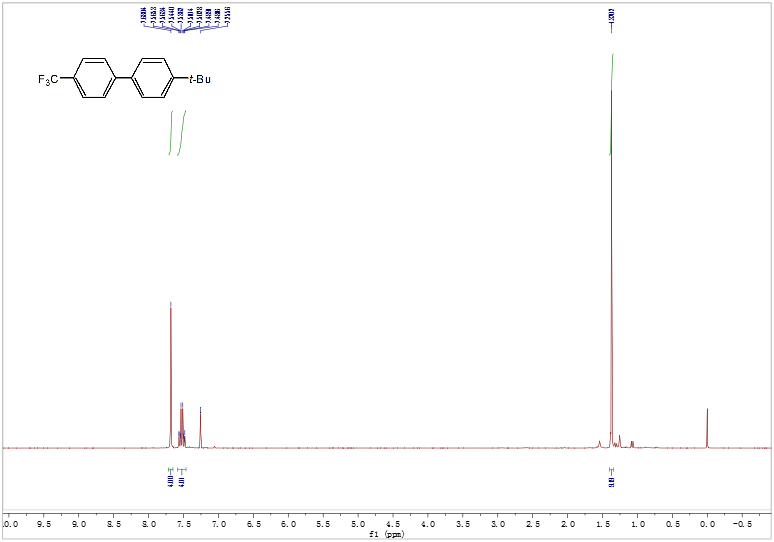
**

**
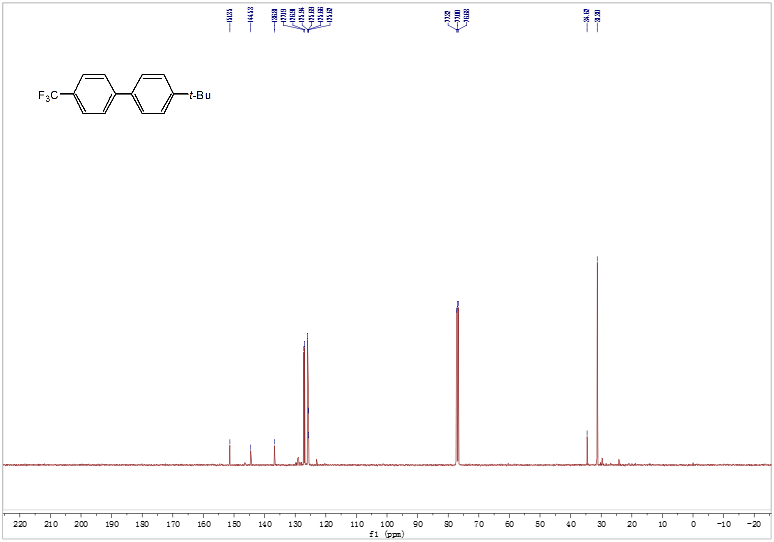
**

**2,2'-Dimethylbiphenyl (4f)**


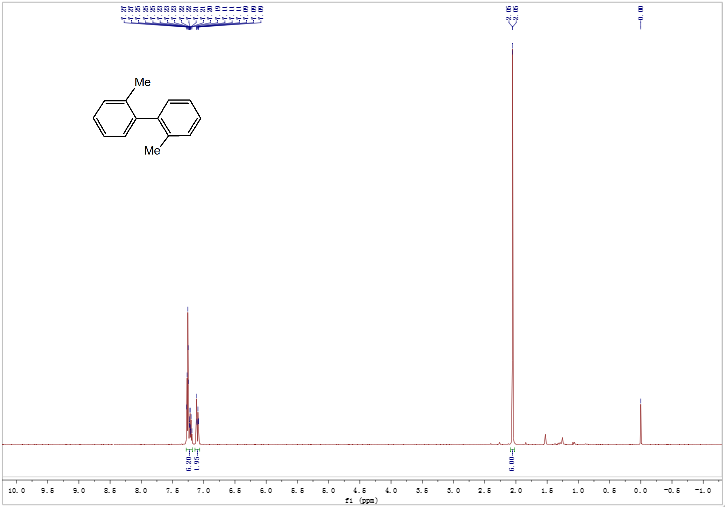


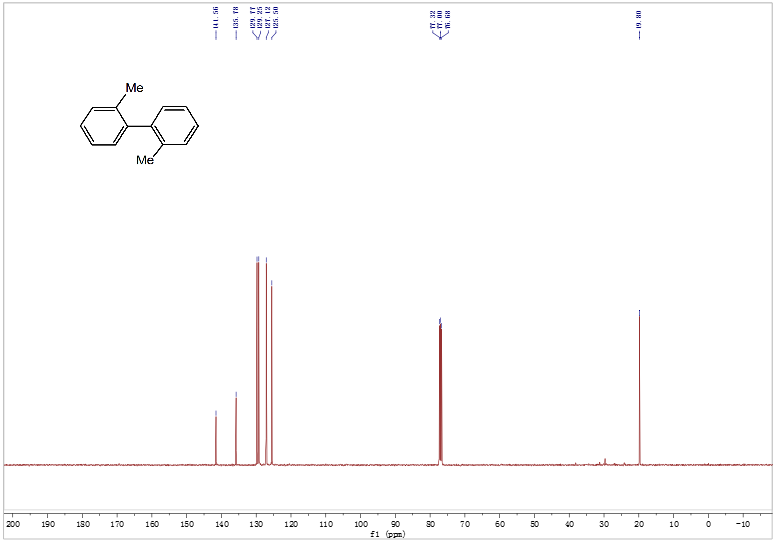


**2,2'-Dimethoxybiphenyl (4g)**


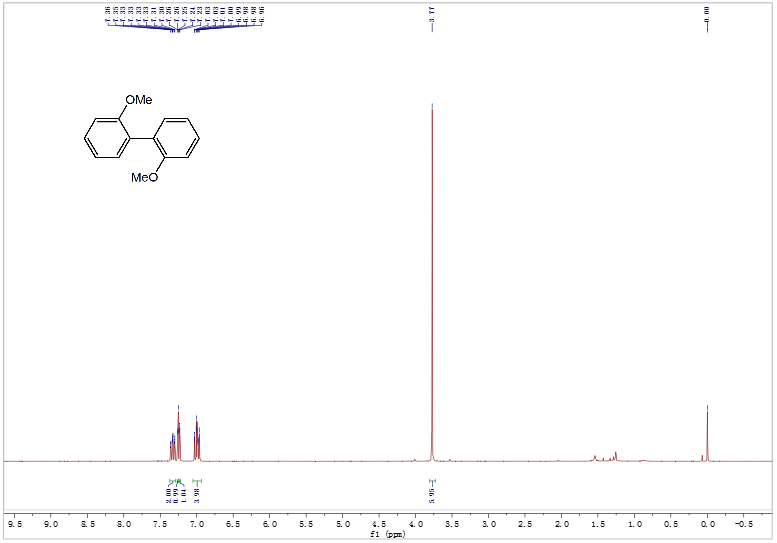


**
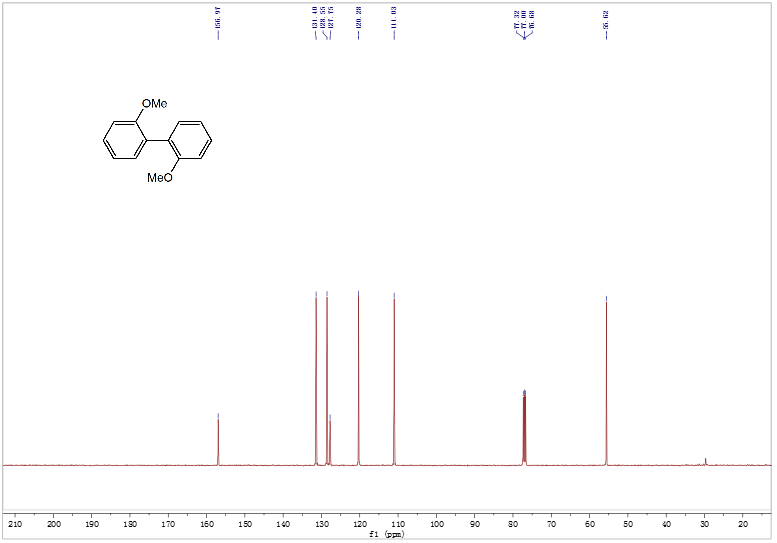
**

**3-[4-(*tert*-Butyl)phenyl]-6-fluoro-pyridine (4h)**

**
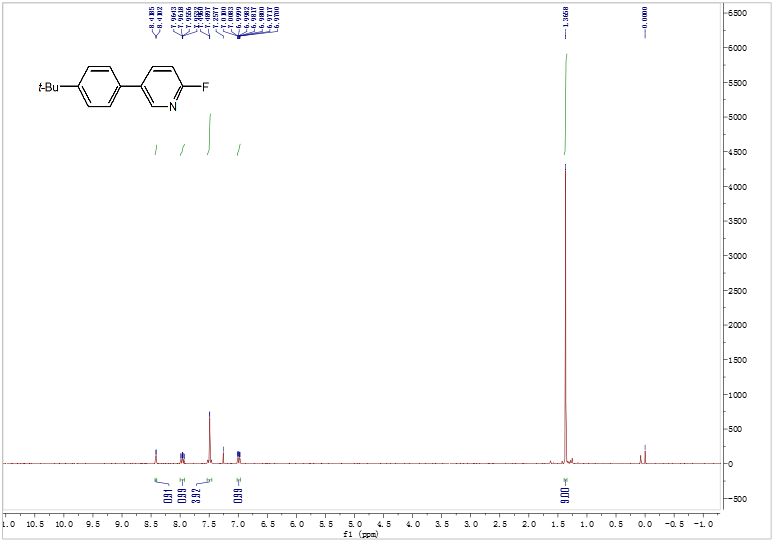
**

**
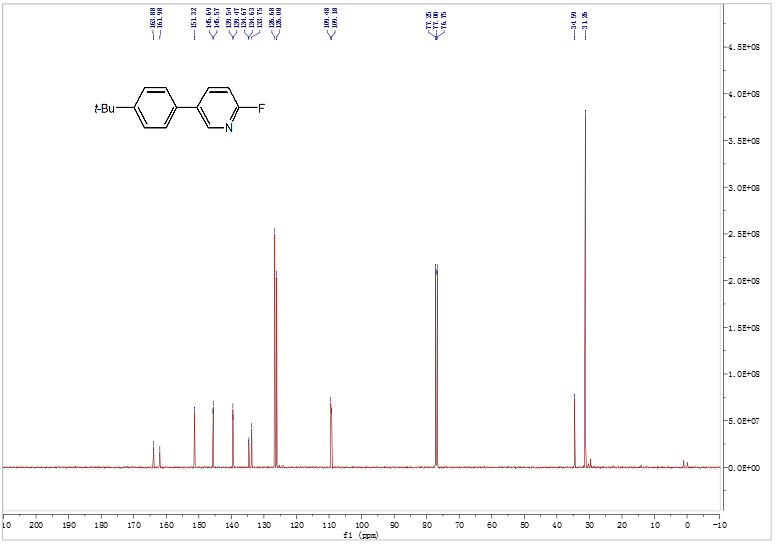
**

**6-(6-Fluoropyridin-3-yl)-1*H*-indole (4i)**

**
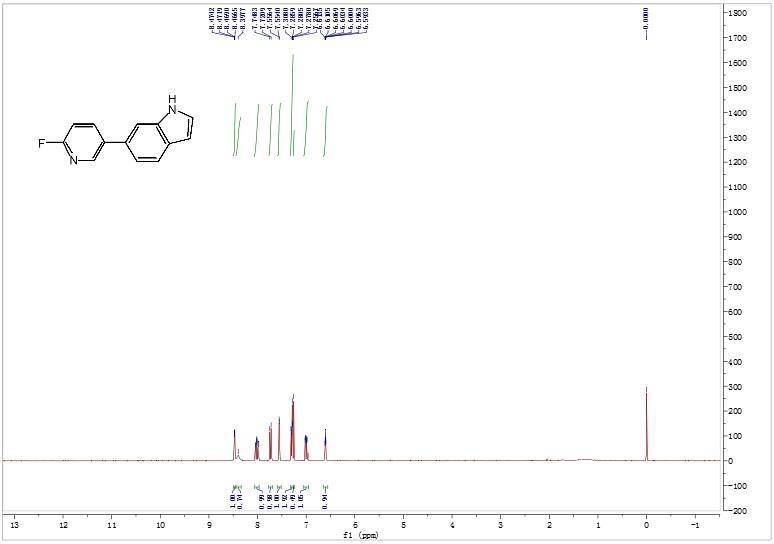
**

**
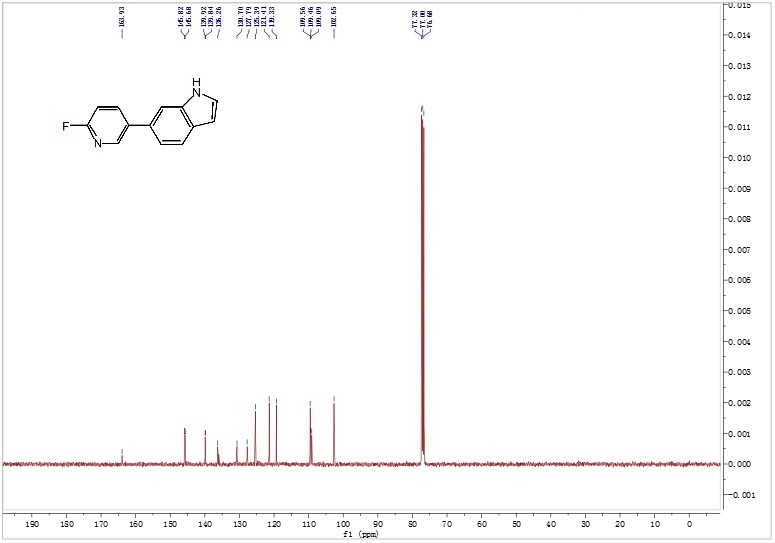
**

**1-Methyl-4-(thiophen-2-yl)-1*H*-pyrazole (4j)**

**
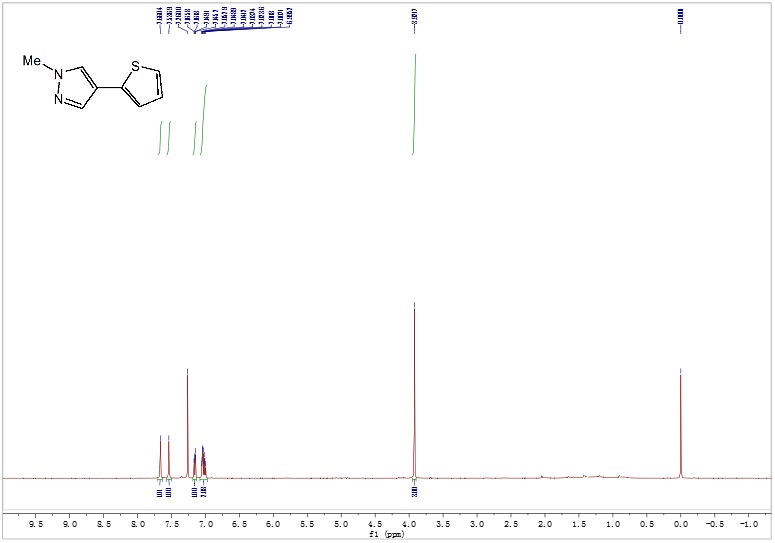
**

**
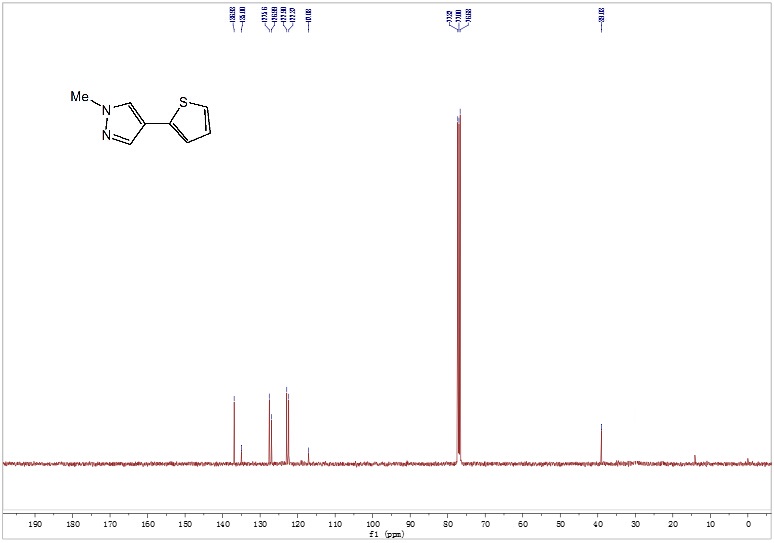
**

**3-Methoxy-4'-(*tert*-butyl)-1,1'-biphenyl (4k)**

**
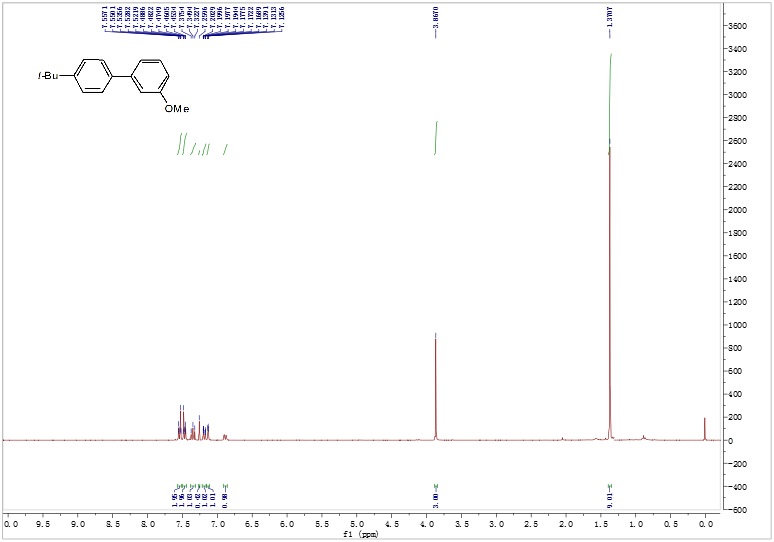
**

**
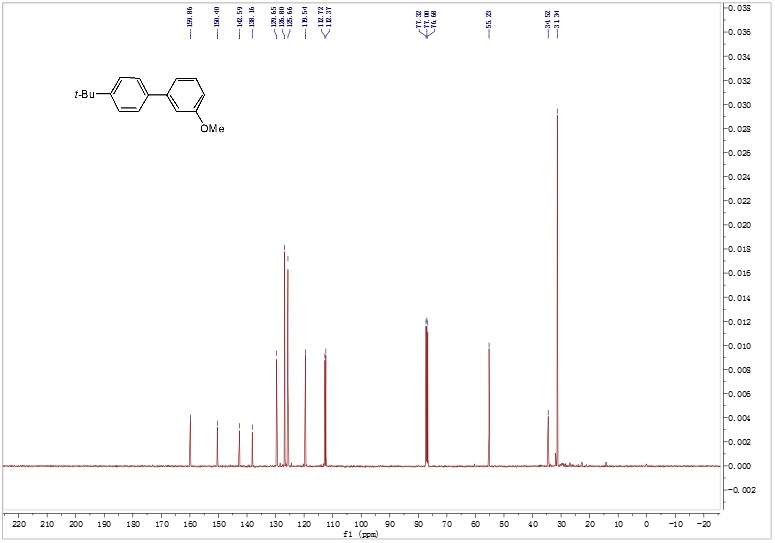
**

**4'-(*tert*-Butyl)-[1,1'-biphenyl]-3-carbonitrile (4l)**

**
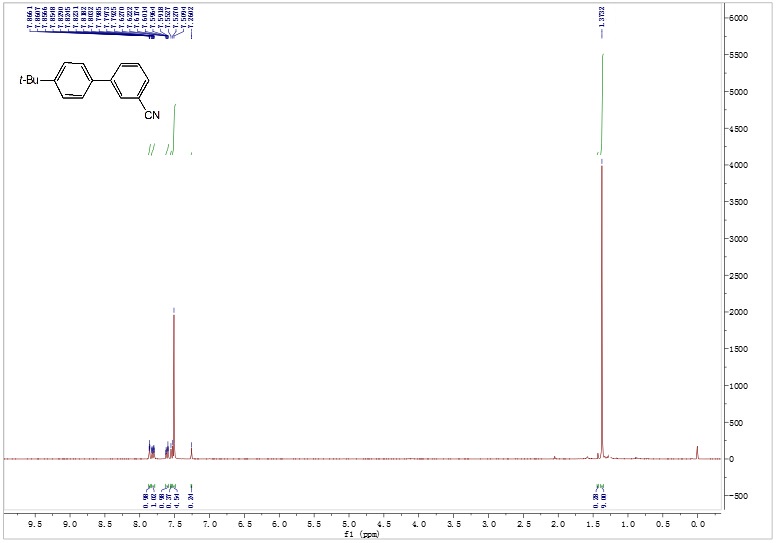
**

**
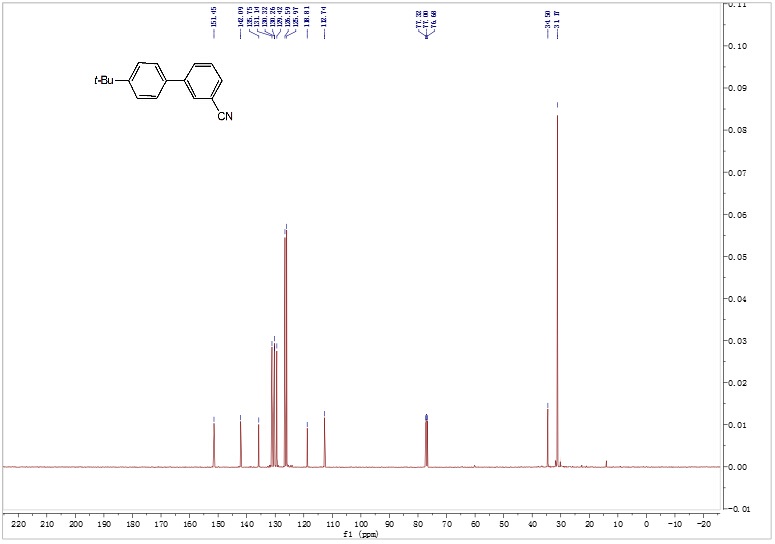
**

**4'-(*tert*-Butyl)-3-nitro-1,1'-biphenyl (4m)**

**
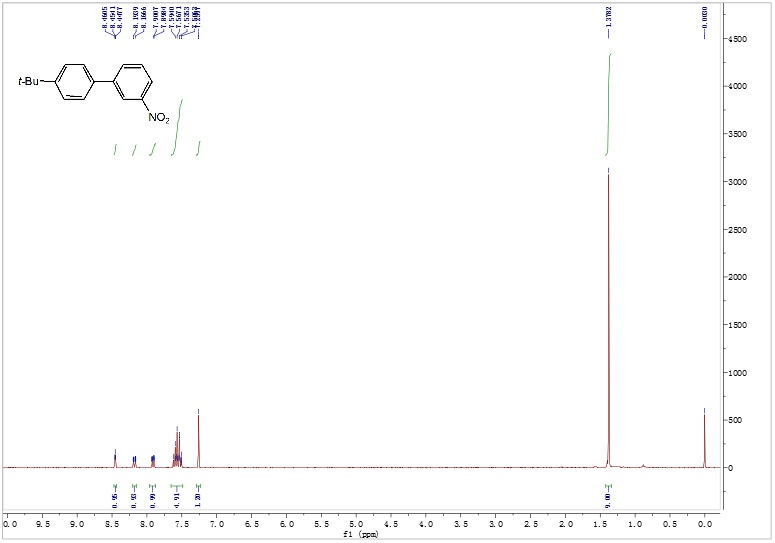
**

**
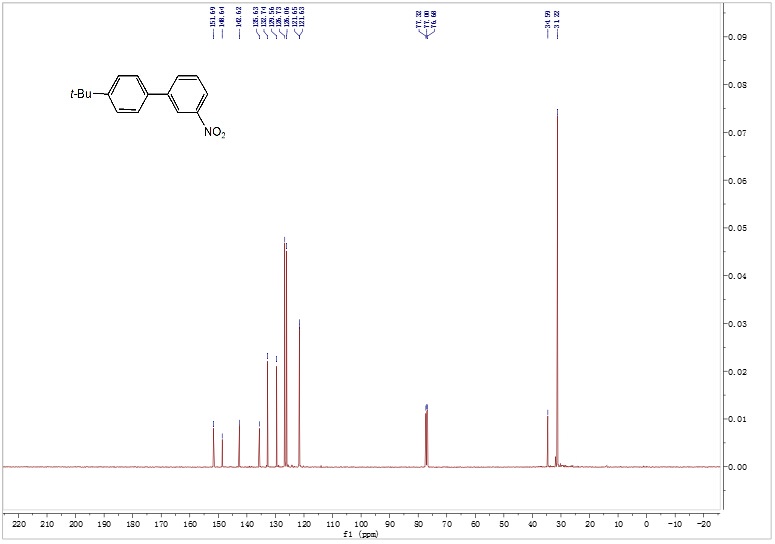
**

**3-[4-(*tert*-Butyl)phenyl]pyridine (4n)**

**
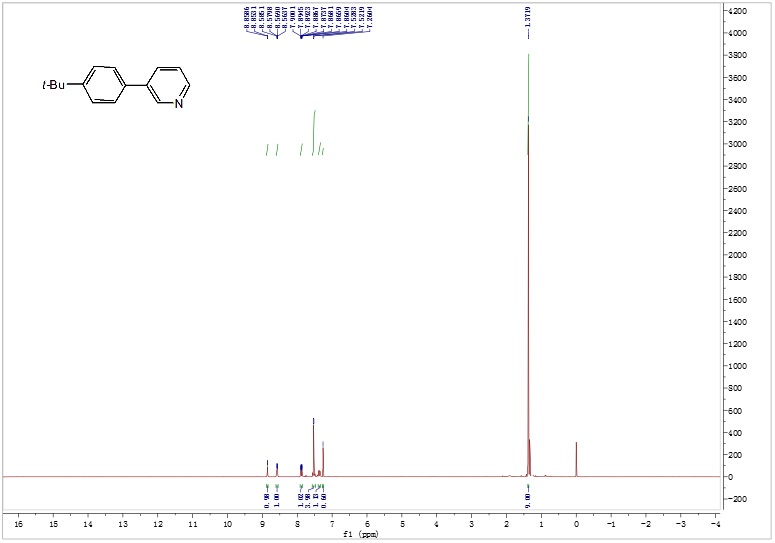
**

**
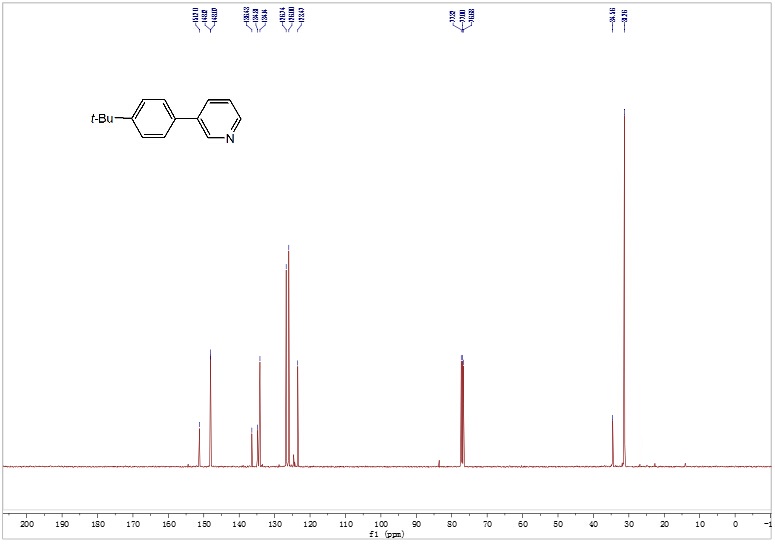
**

**6-[4-(*tert*-Butyl)phenyl]-1*H*-indole (4o)**

**
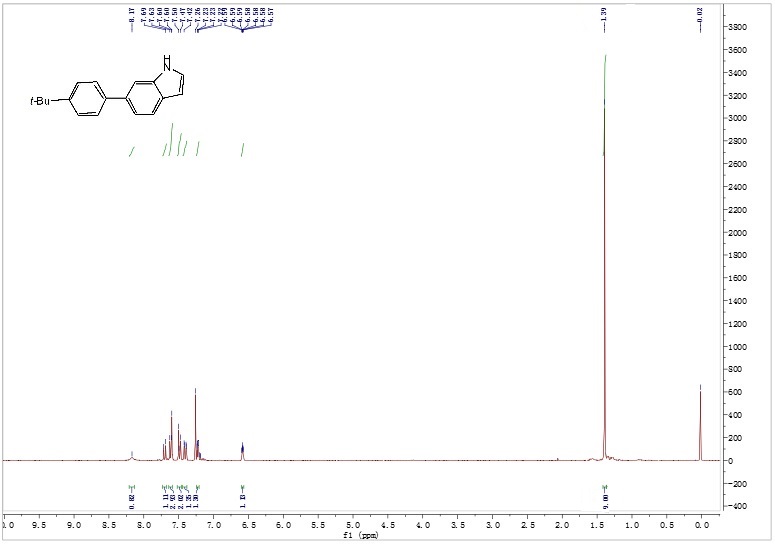
**

**
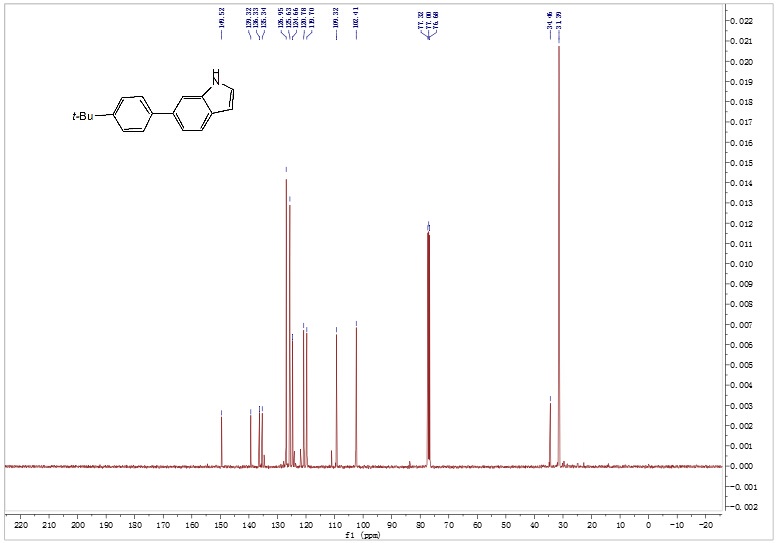
**

**4-[4-(*tert*-Butyl)phenyl]-1-methyl-1*H*-pyrazole (4p)**

**
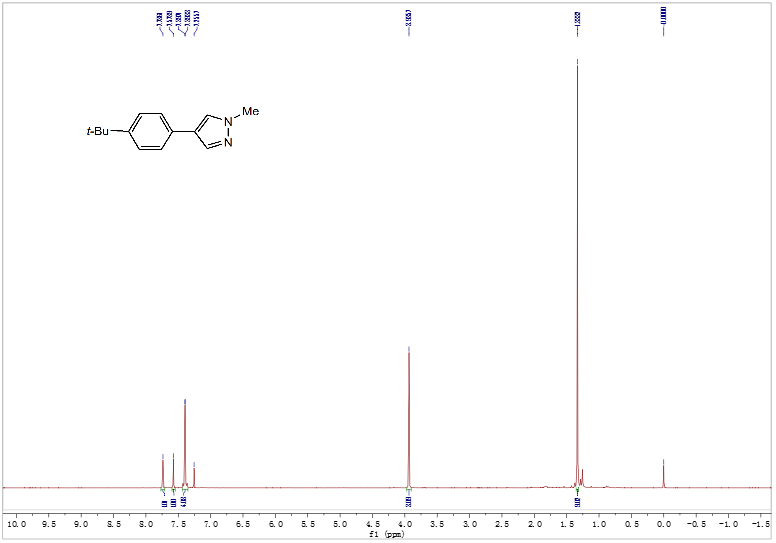
**

**
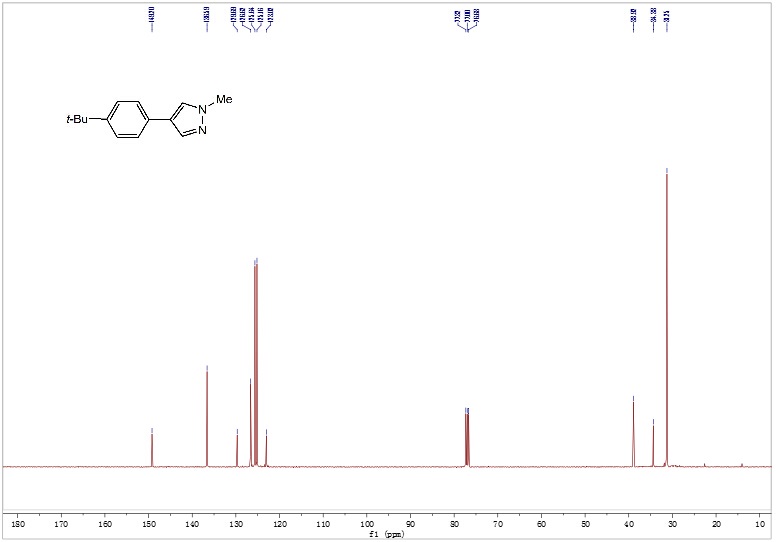
**

**Reference**

1. Molander, G. A.; Cavalcanti, L. N.; Canturk, B.; Pan, P. S.; Kennedy, L. E. [*J. Org. Chem.* **2009**, *74*, 7364-7369](javascript:;).

2. Bruno, N. C.; Tudge, M. T.; Buchwald, S. L. *Chem. Sci.* **2013**, *4*, 916-920.

3. Dzhevakov, P. B.; Topchiy, M. A.; Zharkova, D. A. *Adv. Syn. Cat.* **2016**, *358*, 977-983.

4. Moland, G. A.; Trice, S. L.; Kennedy, S. M. *J. Org. Chem.* **2012**, *77*, 8678-8688.

5. Chen, J. S.; Tanaka, M.; Sahoo, A. K.; Takeda, M.; Yada, A.; Nakao, Y.; Hiyama, T. [*B. Chem. Soc. Jpn*](http://www.medsci.cn/sci/submit.do?id=a4a7954). **2010**, *83*, 554-569.

6. Claudel, S.; Gosmini, C.; Paris, J. M.; Perichon, J. [*Chem. Comm.* **2007**, *35*, 3667-3669](javascript:;).

7. Hu, J. F.; Sun, H. Q.; Cai, W. S.; Pu, X. H.; Zhang, Y. M.; Shi, Z. Z. *J. Org. Chem*. **2016**, *81*, 14-24.

8. Tajuddin, H.; Harrisson, P.; Bitterlich, B.; Collings, J. C.; Sim, N.; Batsanov, A. S.; Cheung, M. S.; Kawamorita, S.; Maxwell, A. C.; Shukla, L.; Morris, J.; Lin, Z. Y.; Marder, T. B.; Steel, P. G. *Chem. Sci.* **2012**, *3*, 3505-3515.

9. Prieto, M.; Zurita, E.; Rosa, E.; Munoz, L.; Lloyd-Williams, P.; Giralt, [*E. J. Org. Chem*. **2004**, *69*, 6812-6820](javascript:;).

10. [Billingsley](http://xueshu.baidu.com/s?wd=author:(Billingsley KL) &tn=SE_baiduxueshu_c1gjeupa&ie=utf-8&sc_f_para=sc_hilight=person), K. L.; [Barder](http://xueshu.baidu.com/s?wd=author:(Barder TE) &tn=SE_baiduxueshu_c1gjeupa&ie=utf-8&sc_f_para=sc_hilight=person), T. E.; [Buchwald](http://xueshu.baidu.com/s?wd=author:(Buchwald SL) &tn=SE_baiduxueshu_c1gjeupa&ie=utf-8&sc_f_para=sc_hilight=person), S. L. *Angew. Chem. Int. Ed.* **2007**, *46*, 5359-5363.

11. Larsen, M. A.; Hartwig, J. F. *J. Am. Chem. Soc.* **2014**, *136*, 4287-4299.

12. Sedelmeier, J.; Ley, S. V.; Baxendale, I. R.; Baumann, M. [*Org. Lett.* **2010**, *12*, 3618-3621](javascript:;).

13. Fairlamb, I. J. S.; Kapdi, A. R.; Lee, A. F. [*Org. Lett.* **2004**, *6*, 4435-4438](javascript:;).

14. Edwards, G. A.; Trafford, M. A.; Hamilton, A. E.; Buxton, A. M.; Bardeaux, M. C.; Chalker, J. M. *J. Org. Chem.* **2014**, *79*, 2094-2104.

15. Bhayana, B.; Fors, B. P.; Buchwald, S. L. *Org. Lett.* **2009**, *11*, 3954-3957.

16. Ren, H.; Xu, Y.; Jeanneau, E. *Tetrahedron*. **2014**, *70*, 2829-2837.

17. Yuan,Y.; Bian,Y. *Appl. Organomet. Chem.*, **2008**, *22*, 15-18.

18. Molander, G. A.; Bernardi, C. R. *J. Org. Chem.*, **2002**, *33*, 8424-8429.

19. Xiong, H.; Hoye, A. T.; Fan, K. H.; Li, X. M.; Clemens, J.; Horchler, C. L.; Lim, N. C.; Attardo, G. *Org. Lett.* **2015**, *17*, 3726-3729.

20. Berman, A. M.; Bergman, R. G.; Ellman, J. A. *J. Org. Chem.* **2010**, *75*, 7863-7868.

21. Chen, Q.; Mao, Z. Q.; Guo, F.; Liu, X. H. *Tetrahedron Lett*. **2016**, *57*, 3735-3738.

22. Nguyen, T.; German, N.; Decker, A. M.; Langston, T. L.; Gamage, T. F.; Farquhar, C. E.; Li, J. X.; Wiley, J. L.; Thomas, B. F.; Zhang, Y. *J. Med. Chem.* **2017**, *60*, 7410-7424.

23. Nguyen, T.; German, N.; Decker, A. M. *J. Med. Chem.* **2017**, *60*, 7410-7424.

24. Gati,W.; Rammah, M. M.; Rammah, M. B.; Couty, F.; Evano, G. *J. Am. Chem. Soc.* **2012**, *134*, 9078-9081.

25. Prieto, M.;  Zurita, E.;  Rosa, E.;  Muñoz, L.; Lloyd-Williams, P.; Giralt, E. *J .Org. Chem.* **2004**, *69*, 6812-6820.

26. Cheng, H.; Wu, Q. Y.; Han, F.; Yang, G. F. *Chinese Chem. Lett.* **2014**, *25*, 705-709.
